# Using deep learning to identify recent positive selection in malaria parasite sequence data

**DOI:** 10.1186/s12936-021-03788-x

**Published:** 2021-06-14

**Authors:** Wouter Deelder, Ernest Diez Benavente, Jody Phelan, Emilia Manko, Susana Campino, Luigi Palla, Taane G. Clark

**Affiliations:** 1 London School of Hygiene & Tropical Medicine, Keppel Street, London, WC1E 7HT UK; 2 Dalberg Advisors, 7 Rue de Chantepoulet, CH-1201 Geneva, Switzerland; 3 Department of Public Health and Infectious Diseases, University of Rome La Sapienza, Rome, Italy

**Keywords:** *Plasmodium falciparum*, *Plasmodium vivax*, Population genomics, Drug resistance, Machine learning, Positive selection

## Abstract

**Background:**

Malaria, caused by *Plasmodium* parasites, is a major global public health problem. To assist an understanding of malaria pathogenesis, including drug resistance, there is a need for the timely detection of underlying genetic mutations and their spread. With the increasing use of whole-genome sequencing (WGS) of *Plasmodium* DNA, the potential of deep learning models to detect loci under recent positive selection, historically signals of drug resistance, was evaluated.

**Methods:**

A deep learning-based approach (called “*DeepSweep”*) was developed, which can be trained on haplotypic images from genetic regions with known sweeps, to identify loci under positive selection. *DeepSweep* software is available from https://github.com/WDee/Deepsweep.

**Results:**

Using simulated genomic data, *DeepSweep* could detect recent sweeps with high predictive accuracy (areas under ROC curve > 0.95). *DeepSweep* was applied to *Plasmodium falciparum* (n = 1125; genome size 23 Mbp) and *Plasmodium vivax* (n = 368; genome size 29 Mbp) WGS data, and the genes identified overlapped with two established extended haplotype homozygosity methods (within-population iHS, across-population Rsb) (~ 60–75% overlap of hits at P < 0.0001). *DeepSweep* hits included regions proximal to known drug resistance loci for both *P. falciparum* (e.g. *pfcrt*, *pfdhps* and *pfmdr1*) and *P. vivax* (e.g. *pvmrp1*).

**Conclusion:**

The deep learning approach can detect positive selection signatures in malaria parasite WGS data. Further, as the approach is generalizable, it may be trained to detect other types of selection. With the ability to rapidly generate WGS data at low cost, machine learning approaches (e.g. *DeepSweep*) have the potential to assist parasite genome-based surveillance and inform malaria control decision-making.

**Supplementary Information:**

The online version contains supplementary material available at 10.1186/s12936-021-03788-x.

## Background

Malaria, caused by *Plasmodium* parasites, is a major global health burden, with an estimated 229 million cases and 409,000 deaths in 2019 alone [1]. *Plasmodium falciparum* causes almost half of all malaria cases, and the majority of deaths are children in sub-Saharan Africa; *Plasmodium vivax* accounts for 65% of malaria cases in Asia and South America [1]. Malaria control involves a combination of case management using diagnosis and treatment, and prevention using insecticide-treated nets, indoor residual spraying, and intermittent preventive treatment.

Resistance to anti-malarial medicines is a threat to the global efforts to control and eliminate malaria. Resistance originates from *Plasmodium* genetic mutations that increase in frequency over time and “sweep” through populations. During the past fifty years, several first-line treatments for *P. falciparum* malaria, including chloroquine and sulfadoxine-pyrimethamine (SP), have been rolled-out and then subsequently replaced due to the emergence of resistance. Recently, resistance to artemisinin has been reported in the form of delayed parasite clearance in Southeast Asia, posing a threat to the current first-line artemisinin-based combination therapy [2, 3]. For *P. vivax*, the spread of resistance to chloroquine, primaquine, mefloquine, and SP has been reported in various regions of the world [4, 5]. The underlying mutations causing resistance for *P. vivax* are less well defined than for *P. falciparum* [4–6].

Protecting and monitoring the efficacy of antimalarial treatments is a top priority for malaria endemic countries. There is a need to not only continuously monitor for drug resistance, which includes clinical reporting, but also to screen the parasite genome for known resistance mutations (e.g. in *P. falciparum*: *pfcrt (PF3D7_0709000)*, *pfdhfr (PF3D7_0417200*)*, pfdhps (PF3D7_0810800)*, *pfmdr1 (PF3D7_0523000),* and *pfkelch13 (PF3D7_1343700*) [3]) and to identify potentially novel loci under putative positive selection. These insights are being facilitated by the characterization of genomic variation using whole-genome sequencing (WGS) across many *Plasmodium* isolates, and the subsequent application of statistical and population genomics methods to detect sweeps. In particular, sweeps can be identified through statistical approaches considering population differentiation, site-frequency spectra, or linkage disequilibrium and extended haplotype homozygosity (e.g. the within population integrated haplotype score (iHS), and the between population ratio (Rsb)) [7]. Whilst these methods have been developed for the human genome [8], they have been applied to *Plasmodium* and identified known genetic mutations contributing to drug resistance [9, 10]. Recently tools have been developed for the efficient computation of these statistics from WGS libraries, such as REHH, SweeD and OmegaPlus [11–13], but they require parameter optimization and their results are sensitive to the SNPs included, population definition, and to the statistical significance thresholds used to make inferences.

In recent years, researchers have explored the possibility of augmenting traditional approaches to the detection of selective sweeps with machine learning methods [14]. To date, sweep detection algorithms have been applied to pre-calculated population genetic statistics (e.g. Tajima’s D, Fay and Wu’s H) [7]. Gradient boosted decision trees and random tree classifiers have been trained on simulated data and applied to human 1000 Genomes Project data [15]. However, these methods do not solve the challenge of defining and calculating the population genetic statistics used as predictors of selection, a task which can be complex and time-consuming, especially when there are multiple sub-populations for cross-comparison. Deep (machine) learning methods may provide a viable alternative, and allow algorithms to learn through a hierarchy of features, where their definition and relationships can be inferred by the algorithm rather than externally defined [16]. The application of neural networks and deep learning has been explored within population genetics [17–19]. More generally, these methods are gaining traction in healthcare and biomedical settings, where enormous amounts of data are being generated, which contain extremely valuable signals and information, at a pace far surpassing what “traditional” methods of analysis can process [19].

The detection of recent positive selection seems amenable to deep learning approaches, where learning to recognize features in raw SNP data, such as the length and shape of shared haplotypes in genes with known sweeps within and between populations, may help to identify sweeps across the genome. The work presented applies a deep learning image-classification approach, which does not require prior extraction or selection of population-genetic statistics, to classify selective sweeps from “haplotypic” images. Using large *P. falciparum* (n = 1125) and *P. vivax* (n = 368) WGS datasets, partitioned into training and validation sets, the analysis shows that a deep learning approach (called “*DeepSweep”*) calibrates well with other haplotype-based methods and other studies, and has the potential to detect novel signatures of positive selection.

## Methods

### Deep learning approach

*DeepSweep* is a deep learning model to detect instances of positive selection. It creates and analyses standardized images of the nearby genomic region around a given SNP. In brief, for each SNP of interest, and across all isolates, *DeepSweep* selects neighbouring SNPs at regularly spaced intervals, and subsequently sorts the remaining genomic matrix in alignment with the longest common haplotype, grouped for each population and for the reference and alternative alleles. The intuition is that SNPs that have undergone recent selective sweeps have a different haplotype structure resulting in distinct images (Additional file 1: Figure S1).

### Model structure

*DeepSweep* uses a convolutional neural network (CNN) architecture, implemented using the Keras library (version 2.2.4) [20] in Python. The model was based on the AlexNet Classifier architecture, widely used for image analysis [21]. Through optimization, it was aimed to fit the smallest sized model (in terms of number of trainable parameters) that showed good predictive performance with low validation loss and high validation accuracy, but also detected features of interest, avoided overfitting, and minimized computational burden. Informally, overfitting is the training of a model that is too specifically tailored to (artefacts in) the training dataset and does not generalize well to unseen data. Statistically, within the framework of the bias–variance trade-off of a model, overfitting occurs where there is excessive variance resulting from an algorithm modelling the random noise in the training data [22]. The approach optimized over various hyper-parameters, including the number of convolutional layers (ranging from 1 to 5 layers), the number of filters (ranging from 2 to 96) and convolutional field sizes (ranging from 3 × 3 to 40 × 40). Regularization techniques (e.g. dropout [22]) were applied to prevent overfitting and support transferability. The model was trained to reduce binary cross-entropy between actual labels and estimated probabilities on images of known- and non-sweeps. The model structure was validated for 500 epochs. The final model has one convolutional layer, two dense layers, four convolutional filters, and a large convolutional field (40 × 9). The haplo-imaging algorithm and the machine learning analyses (Additional file 1: Figures S1, S2) were conducted in Python (version 2.7). The core packages for the machine learning were SnpEff (for annotating effect size) [23], SnpSift (for filtering VCF files) [24], PyVCF (for adjusting and creating VCF files) [25], SciPy and matplotlib (for image manipulation) and Tensorflow (version 1.15).

### Simulated data

Sequence data was generated using SFS_Code software [26], which is a forward population genetic simulator. Simulated data corresponded to four sweep types ((i) recent—strong; (ii) recent—weak; (iii) historic—strong; (iv) partial) and compared to a Wright-Fisher “neutral” setting. The parameter settings are outlined (Additional file 1: Table S1), and lead to plausible scenarios for *Plasmodium* parasites [10]. For each comparison, 160 simulated datasets (128 training; 32 validation) were generated, each dataset with 4 populations of 100 parasite sequences (50% sweep, 50% neutral) and a locus length of at least 1kbp, where the mutation under selection was in the centre of the region. For the combined analysis of the sweep types, 640 simulated datasets (512 training, 128 validation) were used. These data were subsequently transformed into the aforementioned “haplotype images” that serve as input to the image classifier (Additional file 1: Figure S1). These haplo-images showed qualitatively discernible differences in features, with stronger or more recent sweeps leading to more “block-like” features (Additional file 1: Figure S3). The image classifier was trained on the simulated data, and classification accuracy and reduction of binomial loss were estimated. Simulated data was also used to illustrate the impact of changes in a subset of hyperparameters and confirmed that the final model had low validation loss and high validation accuracy (Additional file 1: Table S2).

### Plasmodium sequencing data

Publicly available raw Illumina WGS data for *P. falciparum* (n = 1125) [27] and *P. vivax* (n = 368) [28], representing 11 malaria endemic countries (Additional file 1: Table S3; accession numbers in Additional file 1: Tables S4, S5). All samples were assessed by estMOI software [29] as either monoclonal or polyclonal samples with only a major dominant clone, to minimize the effects on analysis of multiplicity of infection. The *P. falciparum* and *P. vivax* sequences were mapped to the *Pf3D7* (23Mbp) and *PvP01* (29Mbp) reference genomes, respectively, using *bwa-mem* software (version 0.7.12; using default parameter settings) [30]. From the resulting alignments, SNPs and insertions and deletions (indels) were called from the consensus of *GATK* (version 4.1.4.1) [31] and *samtools* (version 1.9) [32] software (using default parameter settings), as applied in previous studies [4, 10]. SNPs were retained if they had < 10% missing alleles and a minor allele count greater than 4. The resulting dataset comprised of parasite genomes of *P. falciparum* (1,125 isolates, 74,757 SNPs) and of *P. vivax* (368 isolates, 126,596 SNPs). The number of missing values was 1,179,202 (2.9%) for *P. vivax* and 649,337 (1.2%) for *P. falciparum*. Missing alleles were imputed using the isolate with the longest shared haplotype around the missing position. An overview of the analytical approach is summarized (Additional file 1: Figure S2). The SnpEff tool (https://pcingola.github.io/SnpEff/) was used to annotate SNP variants and predict their effects on genes.

For *DeepSweep* model training, the presumed positive examples of positive selection are regions surrounding SNPs that are linked to drug resistance with an established scientific literature. For *P. falciparum,* these included regions around established SNPs in *pfcrt* (K76T, I356T; chloroquine)*, pfdhfr* (N51I, C59R, S108N, I164L, S306F)*/pfdhps* (I431V, S436A, A437G, K540E/N, A581G, S613S) (SP)*, pfmdr1* (N86Y; mefloquine, chloroquine)*,* and *pfkelch13* (F446I, Y493H, P574L, R539T, and C580Y; artemisinin) [27]*.* For *P. vivax*, these included regions around some known SNPs in *pvdhps* (A553G, G383A, S382C/A) */ pvdhfr* (N50I, F57I/L, S/K58R, T61M, N117T/S) (putative SP) and *pvmdr1* (F1076L, Y976F, S698G, S513R; putative chloroquine) [4, 6]. This could be considered a relatively small number of training exemplars, which may lead to an increased risk that the implemented machine learning algorithm overfits due to potential artefacts in the training data. Therefore, for each *Plasmodium* species, “leave-one-group-out” cross-validation was implemented to understand the influence of individual training genes, where each single gene of the positive training examples was omitted in turn, with the model trained on the remaining genes [33]. The final model was fit on 80% of the data (split by SNPs), with 20% left as a hold-out set. The *DeepSweep* approach was compared to traditional haplotype-based statistics (iHS [34] and Rsb [35]), as calculated with the REHH package [36].

## Results

### Simulation study

Across the 4 different types of sweep simulations, the predictive accuracy was highest for more recent strong selection (97.1%), followed by weak selection (96.8%) and historic selection (88.2%) and partial selection (86.7%) (Table 1, Additional file 1: Figure S4). The total sensitivity across all sweeps combined was 89.1%, with a specificity of 93.8%, and an overall classification accuracy of 91.4%. The areas under the ROC curve were high for all simulations involving recent selection (> 0.95; maximum 1), consistent with the high predictive ability of *DeepSweep*. The simulation results showed the potential utility of the approach when combining data across populations with common sweeps at difference stages.

**Table 1:** Model performance based on simulated data

	Acc %	Sens %	Spec %	AUC
Stronger selection—recent sweep	97.1	93.8	100	1
Stronger selection—historic sweep	88.2	93.8	83.3	0.858
Weaker selection—recent sweep	96.8	100	93.3	1
Partial sweep	86.7	87.5	85.7	0.951
All sweeps combined	91.4	89.1	93.8	0.944

*Acc* accuracy, *Sens*. Sensitivity, *Spec*. specificity, *AUC* Area under the ROC Curve

### Plasmodium falciparum DeepSweep analysis

The dataset comprised of 1,125 isolates and 74,757 SNPs. Most of these SNPs are in genic regions (76.5%), with 63.0% non-synonymous amino acid changes. Most SNPs have low minor allele frequencies (SNPs with MAF < 1%: 94.6%) (Additional file 1: Figure S5). The image classifier was trained on regions covering the established resistance SNPs in five genes, and found the models validated well using a leave-one-group-out approach. In particular, the overall accuracy was 83.6% (standard deviation 6.0%), where the performance was lower when *pfdhfr* was omitted (75.0%) and was higher when *pfdhps* (92.3%) was left out. One interpretation is that *pfdhfr* is under stronger selection than *pfdhps*, which would be consistent with *pfdhfr* N51I, C59R, S108N, I164L and S306F mutations underpinning key haplotypes underlying SP resistance [37]. The final model was fitted on 80% of the data, with 20% of the data used as a validation set, and demonstrated a strong performance both in terms of classification accuracy and reduction of binomial loss (Additional file 1: Figure S6). The trained classifier was then used to make predictions for the entire dataset of *P. falciparum* SNPs.

**Fig. 1:**
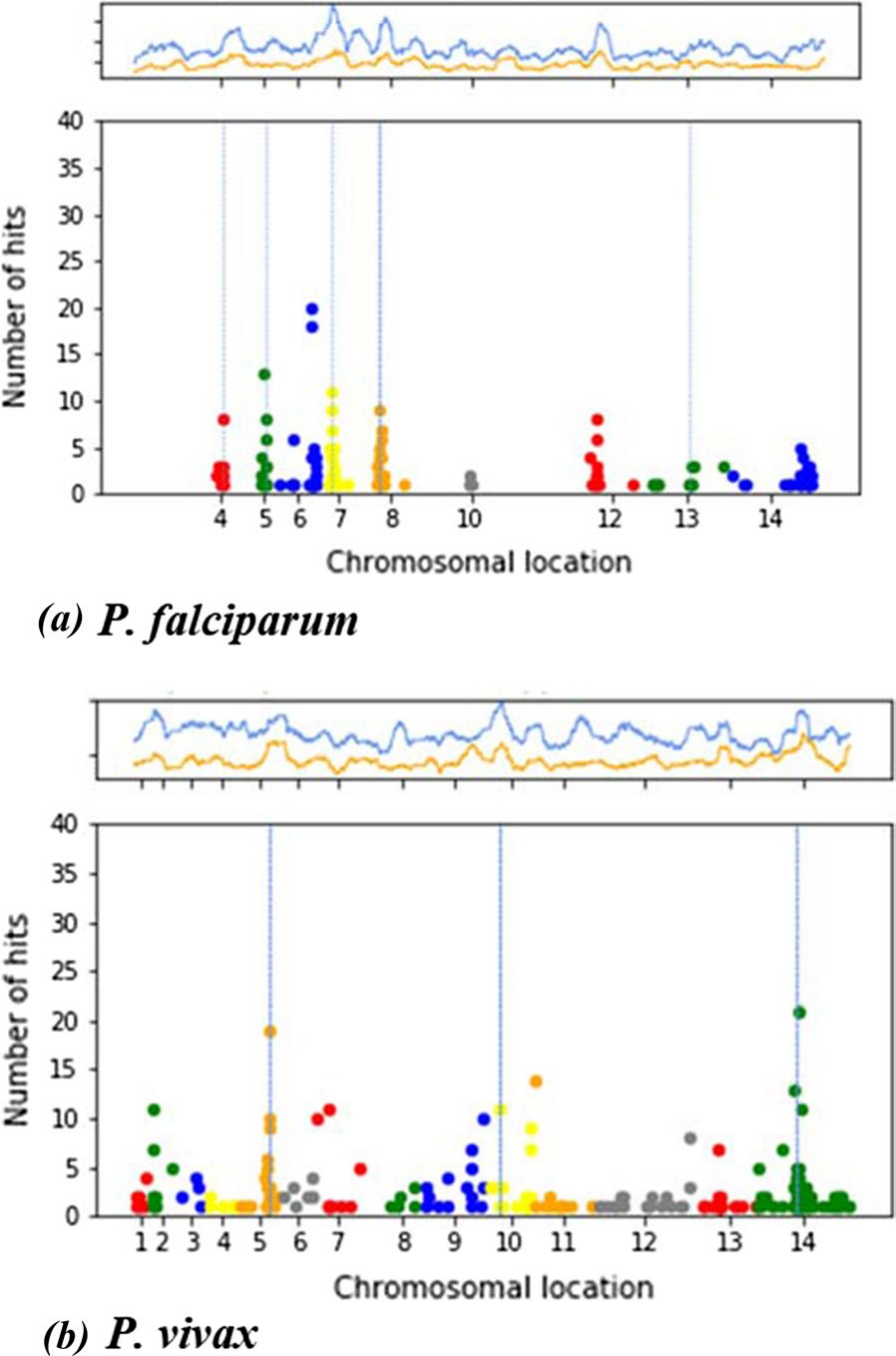
Number of *DeepSweep* Hits per locus across the 14 chromosomes and the relationship to the number of Rsb (top panel; blue line) and iHS (top panel; orange line) hits. **a** *P. falciparum.* Blue line is the running average of Rsb hits (p < 0.0001) over the nearest 100 locations. The orange line is the running average of iHS hits (p < 0.0001) over the nearest 100 locations. The vertical blue lines indicate *pfdhfr* (Chr. 4: 749,001, *pfmdr1* (Chr. 5: 960,020), *pfcrt* (Chr. 7: 404,770), *pfdhps* (Chr. 8: 549,408); *pfkelch13* (Chr. 13: 1,724,817). The tick-marks on the x-axis are chromosomal mid-points. **b** *P. vivax*. Top panel: Blue line is the running average of Rsb hits (p < 0.0001) over the nearest 100 locations. The orange line is the running average of iHS hits (p < 0.0001) over the nearest 100 locations. The vertical blue lines indicate *pvdhfr* (Chr. 5), *pvmdr1* (Chr. 10), *pvdhps* (Chr. 14). The tick-marks on the x-axis are chromosomal mid-points

The deep learning model identified 387 SNPs in 160 genes (or ~ 2.9% of genes) as putatively under positive selection pressure in the wider dataset (Fig. 1). Further analysis focused on the subset of 11 genes that have > 6 hits (Table 2; see Additional file 1: Table S6 for the 26 genes with > 3 SNPs). Several peaks were in the vicinity of known drug-resistance genes in the training set, with nearby genes likely to be swept along (e.g. on *pfdhfr* on chromosome 4, *pfmdr1* on chromosome 5, *pfcrt* on chromosome 7, *pfdhps* on chromosome 8 and *pfkelch13* on chromosome 13). There is an additional peak on chromosome 6 that includes *Pk4* (*PF3D7_*0628200) and the HECT domain (*PF3D7_*0628100). Transcription of *Pk4* has been related to artemisinin-induced latency [38], and the HECT domain is thought to alter quinine and quinidine response, and likely co-selected with *pfcrt* [39]. There is a small peak on chromosome 10 (*PF3D7_1013500*) in the close vicinity of the gene encoding the autophagy-related protein 18 (*PF3D7_1012900*), which has been associated with artemisinin resistance. There is a peak on chromosome 12 (*PF3D7_1223500*) which has been putatively associated with SP resistance [40]. Smaller peaks were observed on chromosome 14 around *PF3D7_1462400,* which has been associated with chloroquine resistance [41].

**Table 2:** *Plasmodium falciparum* loci identified by *DeepSweep* (DS; with > 6 SNPs)

Chr	Gene ID (*PF3D7_)*	DS hits	iHS hits	Rsb hits
6	627800*	20	11	39
6	628100*	18	1	30
5	522400**	13		8
7	709100**	11		38
7	708200**	9		14
8	809600**	9	3	29
4	417400**	8		37
5	522900**	8		
12	1223500*	8		11
7	709300**	7		46
8	811200**	7		11

*Chr* Chromosome; iHS and Rsb counts defined as the number of SNPs in a gene that have an |iHS| or |Rsb| score with a p-value < 0.0001; *pfdhfr* (Chr. 4: 749,001, *pfmdr1* (Chr. 5: 960,020), *pfcrt* (Chr. 7: 404,770), *pfdhps* (Chr. 8: 549,408; * previously identified; ** close to known gene

### Plasmodium vivax DeepSweep analysis

The dataset comprised of 368 isolates and 126,596 SNPs. Most of these SNPs are in genic regions (77.6%), with 42.5% non-synonymous amino acid changes. Many SNPs have low minor allele frequencies (SNPs with MAF < 1%: 77.6%) (Additional file 1: Figure S5). The image classifier was trained on the sixteen SNP mutations in the three genes. Using a leave-one-group-out validation approach, the overall accuracy was 79.7% (standard deviation 17.6%), and the performance was lower when *pvmdr1* was omitted (57.1%) and was higher when *pvdhfr* was left out (100%). This difference is consistent with *pvmdr1* residues being strongly associated with chloroquine resistance [5] and, although, *pvdhfr* may contribute to SP drug resistance, there are very few published studies that associate genotypes of this locus with anti-folate susceptibility phenotypes [6]. As with *P. falciparum,* the trained model had strong performance both in terms of classification accuracy and reduction of binomial loss (Additional file 1: Figure S6). The model identified 577 hits in 237 genes (or ~ 4.3% of genes) as putatively under positive selection pressure in the wider dataset (Fig. 1). Further analysis focused on the subset of 19 genes that have > 6 hits (Table 3; see Additional file 1: Table S7 for the 35 genes with > 3 SNPs). Several loci are near the training genes (*pvdhfr* on chromosome 5, *pvmdr1* on chromosome 10, *pvdhp*s on chromosome 14). Further, there was a peak around the gene encoding for the multi-drug associated protein 1 (*pvmrp1*), which is a putative resistance candidate [4]. On chromosome 7, there was a peak around a gene coding for cysteine repeat modular protein 1, which is expressed in both vertebrate and mosquito hosts for host tissue targeting and invasion. This locus has been identified as presenting high population differentiation and under directional selective pressure in South America [4]. Finally, there was a larger region that was identified on chromosome 14, which contains *pvdhps* and a number of other genes that have been found in other analyses [4].

**Table 3:** *Plasmodium vivax* loci identified by *DeepSweep* (DS; with > 6 SNPs)

Chr	Gene ID (*PVP01_)*	DS Hits	iHS hits	Rsb hits
14	1430700	21		
5	526800**	19	4	12
11	1101300	14		2
14	1428700**	13	1	5
2	202000	11		4
7	709800	11		0
10	1011000**	11		1
14	1432900	11	1	1
5	526400**	10	1	12
7	701100	10		8
9	948800	10		5
5	526300**	9	2	4
10	1034400	9		
12	1271500	8		
2	203000*	7		33
9	939900	7	2	1
10	1033900	7		
13	1317300	7		15
14	1418100	7		1

*Chr* Chromosome, iHS and Rsb Counts defined as the number of SNPs in a gene that have an iHS or Rsb score with a p-value < 0.0001; *pvdhfr* (Chr. 5), *pvmdr1* (Chr. 10), *pvdhps* (Chr. 14); * previously identified; ** close to known gene

### Comparison with established positive selection approaches

An analysis using the established REHH approach was performed, which involved the calculation of the integrated haplotype score (iHS) within populations and the associated Rsb values between pairs of populations (Additional file 1: Tables S8, S9). Although the REHH and *DeepSweep* methods have a different ranking of the strongest hits, there was an overall positive correlation between the number of hits from Rsb and *DeepSweep* (Pearson correlation: *P. falciparum* 0.49, *P. vivax* 0.20; Additional file 1: Figure S7). However, *DeepSweep* detected several novel loci that were not identified by REHH. These included loci on chromosomes 6 (*PF3D7_0611800*), 8 (*PF3D7_0811600*) and 14 (*PF3D7_1461800*) for *P. falciparum* (Additional file 1: Table S6)*,* and on chromosomes 6 (PIR protein), 7 (cysteine repeat modular protein) and chromosome 14 for *P. vivax* (Additional file 1: Table S7). *PF3D7_0611800* has been linked to increased cytoadherence [42]*, PF3D7_0811600* has previously been linked to SP resistance [40] and the genes coding for the PIR protein and the cysteine repeat protein have been associated with immune response and host invasion [43, 44]. There were several loci that were detected by EHH methods but not by *DeepSweep* (Additional file 1: Tables S8, S9). Some of the top hits included genes that are linked to immune response and host invasion (e.g. *PF3D7_1133400* AMA1, *PF3D7_1335100* MSP7). Other hits are housekeeping genes that are less likely to be under selective pressure (e.g. *PF3D7_0731800* (alpha/beta hydrolase), *PF3D7_1475900* (KELT protein), *PVP01_0202900* (18S) and *PVP01_1003700* (PPT)).

## Discussion

The application of whole genome sequencing (WGS) is gaining traction across malaria endemic countries. With the resulting development of *Plasmodium* parasite genomic databases (“big data”), there is an opportunity for the implementation of machine learning methods to inform disease control. The detection of genomic signatures of selective sweeps resulting from the spread of mutations associated with anti-malarial drug resistance is one application of WGS data. This work presents a supervised (deep) learning approach (*DeepSweep)*, which after being trained on haplotypic “images” of established drug resistance genes in *P. falciparum* and *P. vivax* parasites, resulted in the identification of loci known to be under recent positive selection. Whilst the strength of sweep signals per locus found by *DeepSweep* correlated with established EHH methods (e.g. between population Rsb), the machine learning approach has the advantage of not requiring a rigid definition and calculation of population-genetic statistics, incorporating information within and across populations, and relatively lower requirements for the pre-processing of raw SNP data. Like other machine learning approaches, it has the potential to scale up to large numbers of samples, and is parallelizable across genomic regions, thereby making it a potentially useful “big data” tool. In the absence of sufficient computational power, it is possible to develop sampling strategies that can select the subset of the data and samples that contain the highest density of information relevant to *DeepSweep*. Different model structures were assessed, but performance could be improved by further fine tuning of model hyperparameters (e.g. the number and size of the convolutional filters).

*DeepSweep* detected a set of loci not detected by the EHH methods, potentially because a deep learning approach can holistically incorporate information from the raw SNP data, which could be fragmented across separate populations and genomic windows, for the calculation of population-genetic statistics. Indeed, the simulation study demonstrated the potential of including haplo-images with not only single, but multiple populations, to allow the algorithm to take advantage of features that are common across regions and be robust to different stages of the sweeps. However, *DeepSweep* does require “representative” positive training examples, and in the context applied, assumes that the training drug resistance related loci have undergone or are undergoing selective sweeps in some of the populations. This assumption is not unrealistic given that some antimalarial drugs have been rolled out in different populations at different times resulting in differential stages of selective sweeps [40]. The *DeepSweep* and EHH approaches, as well as alternative methods (e.g. HaploPS [45]), can be considered complementary and could be run in parallel. However, as these approaches will increasingly use WGS, there are general challenges that affect variant-calling and ascertainment (e.g. extreme genome GC content), which can impact on the density and accuracy of genomic variant inputs, as well as the final population genomic analysis. Typically, WGS analysis leads to a dense set of well supported variants in robust genomic regions, with the application of calling algorithms incorporating information on known high quality polymorphisms [6]. Further, highly variable or problematic regions, such as *var* genes in *P. falciparum*, are typically removed from analysis [46]. In general, *DeepSweep* appeared to perform well across different GC content settings (*P. falciparum* 19%, *P. vivax* 58%), as well as in a simulated data setting which did not impose any constraint on GC content. However, in general, it is important to evaluate the quality of genomic variants used in an analysis. A further consideration is that most approaches use haplotype data, which in the human context require phasing from genotypes. Whilst the *Plasmodium* life cycle involves haploid asexual stages, complex clinical infections can complicate and confound population genetic analyses, and therefore analysis was restricted to infections with a dominant clone. However, it may be possible to extend *DeepSweep* to process individual parasite sequences for samples with multiplicity of infection. Irrespective, any novel loci identified should be confirmed through functional work [47]. Further, complementary methods that look at isolate relatedness, as determined by identity by descent (e.g. IsoRelate [48]), could also be implemented. New loci detected by *DeepSweep* that were not identified by other methods (e.g. on chromosomes 6, 8 and 14 for *P. falciparum* and on chromosomes 6, 7 and 14 for *P. vivax*) provide interesting candidates for confirmation studies.

A potential future opportunity is to apply models across species, for example, to detect *P. falciparum* loci after being trained on *P. vivax* signatures, and vice-versa. Such an application could assist to detect regions where drug resistance loci are unknown or less established, such as *P. vivax*. However, the impacts of differences in sample size and degree of polymorphism between species need to be considered. Relatedly, “real data” was used for training, but an alternative may be to use coalescent or forward-in-time simulation to create positive and negative labelled exemplars. However, there is a risk that images might not be representative of actual selective sweeps in nature. The deep learning algorithm has applications beyond positive selection, including for other evolutionary signatures (e.g. balancing selection) or application to other organisms (e.g. mosquitoes and humans).

## Conclusions

The *DeepSweep* approach and the wider application of deep learning using genomic images constitutes a novel approach that shows promising results. It provides a robust, accessible and scalable approach for the identification of genomic regions under positive selection, and could assist with detecting established and new types of drug resistance. Thereby, providing insights into transmission dynamics and informing malaria control decision-making.

## Supplementary Information

**Additional file 1**: **Table S1**. Simulation parameters for the data generation using SFS_Code software. **Table S2**. Performance of Convolutional Neural Network (CNN) model structures on simulated datasets. **Table S3**. Sample origin by geographic location. **Table S4**. The 1,125 high-quality P. falciparum isolates used in this study. **Table S5**. The 368 high-quality P. vivax isolates used in the study. **Table S6**. Plasmodium falciparum loci identified by DeepSweep (DS; with >3 SNPs). **Table S7**. Plasmodium vivax loci identified by DeepSweep (DS; with >3 SNPs). **Table S8**. Plasmodium falciparum loci with the most iHS and Rsb hits. **Table S9**. Plasmodium vivax loci with the most iHS and Rsb hits. **Figure S1**. The creation of haplo-images. **Figure S2**. Workflow. **Figure S3**. Exemplar images of simulated isolates undergoing different types of sweeps or neutral evolution. **Figure S4**. Model performance on simulated datasets. **Figure S5**. Distribution of the minor allele frequencies across the SNPs. **Figure S6**. Model performance for Plasmodium falciparum and P. vivax on training and validation datasets. **Figure S7**. Relationship between -log10 p-value of Rsb hits and number of DeepSweep hits.

## Data Availability

The WGS data is available from the European Nucleotide Archive (ENA) (see Additional file 1: Table S4, S5 for project accessions). Computing code is available from https://github.com/WDee/Deepsweep.

## Abbreviations

AUC: Area under the ROC curve
CNN: Convolutional Neural Network
EHH: Extended Haplotype Homozygosity
Indels: Insertions and Deletions
iHS: Integrated Haplotype Score
ROC: Receiver Operating Characteristic
SNP: Single Nucleotide Polymorphism
SP: Sulfadoxine-Pyrimethamine
WGS: Whole Genome Sequencing
