# Supplementary material for: Using deep learning to identify recent positive selection in malaria parasite sequence data
Source: Malar J. 2021 Jun 14;20:270. doi: 10.1186/s12936-021-03788-x (PMC8201710; doi:10.1186/s12936-021-03788-x)

S1 Table

Simulation parameters for the data generation using SFS_Code software

Scenario	No. of Simulated datasets*	Selection Coefficient	Time window***
Stronger selection – recent sweep	80	2000	0.20
Stronger selection – historic sweep	80	2000	0.40
Weaker selection – recent sweep	80	400	0.20
Partial sweep**	80	100	0.15
Neutral	80 x 4	0	0.20

* Each simulated dataset consisted of 4 populations of 100 individual parasites (50% sweep; 50% neutral), and a locus length of 1kbp, with the mutation inserted at position 500. The mutation rate per site was set to 0.01 and the recombination rate per site was 0.02; ** The partial sweeps were created with rejection sampling, where only sweeps that had a derived allele frequency of between 33% and 80% were kept; *** The window is the time between the introduction of the mutation and sampling expressed in 2N generations, with N being the population size.

S2 Table

Performance of Convolutional Neural Network (CNN) model structures on simulated datasets

Model	Description of model and changes*	Trainable parameters	Validation Loss**	Validation Accuracy (%)
1	Model used in our study (one convolutional layer with 4 filters, with respective filter size of (40,9) followed by two drop-out and dense layers with ReLu activation)	4,525	0.33	93.8
2	Two convolutional layers with 4 filters in each layer, with respective kernel size of 11 and 5	78,277	0.42	75.0
3	Three convolutional layers with 4 filters in each layer, with respective kernel sizes of 11, 5 and 3	71,705	0.49	62.5
4	Increase of number of convolutional filters to 8	15,193	0.26	87.5
5	Decrease of number of convolutional filters to 2	1,495	0.59	68.8
6	Increase of drop-out rate to 0.4 (from 0.2)	4,525	0.32	87.5
7	Decrease of drop-out rate to 0.1 (from 0.2)	4,525	0.48	75.0

* Model 1 is the final model used across our datasets, and Models 2 - 7 are deviations from this; ReLu: Rectified Linear Unit. ** Validation loss as measured by binary cross-entropy. The performance (validation loss, validation accuracy) was measured by training on 64 simulated isolates with the same characteristics as other simulations (e.g. undergoing different forms of sweeps as well as neutral evolution) with all performance metrics measured on a validation set of 16 isolates.

S3 Table

Sample origin by geographic location

**Country**	***P. falciparum* N***	***P. falciparum* %**	***P. vivax* N****	***P. vivax* %**
Cambodia	351	31.2	32	8.7
Malawi	221	19.6	-	
Ghana	202	18.0	-	
Vietnam	187	16.6	-	
Thailand	164	14.6	128	34.8
Peru	-	-	58	15.8
Malaysia	-	-	50	13.6
Colombia	-	-	30	8.2
Papua New Guinea	-	-	26	7.1
Mexico	-	-	20	5.4
Ethiopia	-	-	24	6.5
Total	1,125	100	368	100

* see **S4 Table** for a list of sequence data accession numbers

** see **S5 Table** for a list of sequence data accession numbers

S4 Table. The 1,125 high-quality *Plasmodium falciparum* isolates used in this study

Country	Identifier	Country	Identifier	Country	Identifier	Country	Identifier
Cambodia	ERS009721	Cambodia	ERS014167	Cambodia	ERS028699	Cambodia	ERS032695
Cambodia	ERS010057	Cambodia	ERS014168	Cambodia	ERS028700	Cambodia	ERS032696
Cambodia	ERS010059	Cambodia	ERS014169	Cambodia	ERS028701	Cambodia	ERS032697
Cambodia	ERS010062	Cambodia	ERS014170	Cambodia	ERS028702	Cambodia	ERS045932
Cambodia	ERS010066	Cambodia	ERS014171	Cambodia	ERS028703	Cambodia	ERS045933
Cambodia	ERS010067	Cambodia	ERS014172	Cambodia	ERS028704	Cambodia	ERS045934
Cambodia	ERS010216	Cambodia	ERS014173	Cambodia	ERS028705	Cambodia	ERS045935
Cambodia	ERS010217	Cambodia	ERS017698	Cambodia	ERS028706	Cambodia	ERS045936
Cambodia	ERS010318	Cambodia	ERS017699	Cambodia	ERS028707	Cambodia	ERS045937
Cambodia	ERS010319	Cambodia	ERS017700	Cambodia	ERS028708	Cambodia	ERS045938
Cambodia	ERS010320	Cambodia	ERS017702	Cambodia	ERS028709	Cambodia	ERS045939
Cambodia	ERS010321	Cambodia	ERS017705	Cambodia	ERS028710	Cambodia	ERS050859
Cambodia	ERS010322	Cambodia	ERS017706	Cambodia	ERS028712	Cambodia	ERS050860
Cambodia	ERS010323	Cambodia	ERS017707	Cambodia	ERS028713	Cambodia	ERS050865
Cambodia	ERS010324	Cambodia	ERS023736	Cambodia	ERS028714	Cambodia	ERS050866
Cambodia	ERS010325	Cambodia	ERS023737	Cambodia	ERS028715	Cambodia	ERS050870
Cambodia	ERS010327	Cambodia	ERS023738	Cambodia	ERS028716	Cambodia	ERS050872
Cambodia	ERS010330	Cambodia	ERS023739	Cambodia	ERS028717	Cambodia	ERS050885
Cambodia	ERS010331	Cambodia	ERS023740	Cambodia	ERS028718	Cambodia	ERS050890
Cambodia	ERS010332	Cambodia	ERS023741	Cambodia	ERS028719	Cambodia	ERS052777
Cambodia	ERS010333	Cambodia	ERS023742	Cambodia	ERS028720	Cambodia	ERS052778
Cambodia	ERS010334	Cambodia	ERS023743	Cambodia	ERS028721	Cambodia	ERS052781
Cambodia	ERS010335	Cambodia	ERS023744	Cambodia	ERS028722	Cambodia	ERS052784
Cambodia	ERS010336	Cambodia	ERS023745	Cambodia	ERS028723	Cambodia	ERS052785
Cambodia	ERS010337	Cambodia	ERS023746	Cambodia	ERS028724	Cambodia	ERS052790
Cambodia	ERS010346	Cambodia	ERS023747	Cambodia	ERS028725	Cambodia	ERS071770
Cambodia	ERS010511	Cambodia	ERS023748	Cambodia	ERS032018	Cambodia	ERS071771
Cambodia	ERS010516	Cambodia	ERS023749	Cambodia	ERS032022	Cambodia	ERS071773
Cambodia	ERS010590	Cambodia	ERS023750	Cambodia	ERS032026	Cambodia	ERS071774
Cambodia	ERS010592	Cambodia	ERS024123	Cambodia	ERS032029	Cambodia	ERS071776
Cambodia	ERS010598	Cambodia	ERS025100	Cambodia	ERS032037	Cambodia	ERS071777
Cambodia	ERS010669	Cambodia	ERS025257	Cambodia	ERS032050	Cambodia	ERS071778
Cambodia	ERS010672	Cambodia	ERS025258	Cambodia	ERS032060	Cambodia	ERS071779
Cambodia	ERS010673	Cambodia	ERS025259	Cambodia	ERS032109	Cambodia	ERS071781
Cambodia	ERS010779	Cambodia	ERS025262	Cambodia	ERS032135	Cambodia	ERS071782
Cambodia	ERS010786	Cambodia	ERS025263	Cambodia	ERS032243	Cambodia	ERS071784
Cambodia	ERS013843	Cambodia	ERS025265	Cambodia	ERS032246	Cambodia	ERS071787
Cambodia	ERS013844	Cambodia	ERS025266	Cambodia	ERS032249	Cambodia	ERS071791
Cambodia	ERS014154	Cambodia	ERS025267	Cambodia	ERS032255	Cambodia	ERS071794
Cambodia	ERS014155	Cambodia	ERS025268	Cambodia	ERS032258	Cambodia	ERS071796
Cambodia	ERS014156	Cambodia	ERS025269	Cambodia	ERS032683	Cambodia	ERS071797
Cambodia	ERS014157	Cambodia	ERS025271	Cambodia	ERS032686	Cambodia	ERS071799
Cambodia	ERS014158	Cambodia	ERS025272	Cambodia	ERS032687	Cambodia	ERS071800
Cambodia	ERS014159	Cambodia	ERS025273	Cambodia	ERS032688	Cambodia	ERS071809
Cambodia	ERS014161	Cambodia	ERS025274	Cambodia	ERS032689	Cambodia	ERS071812
Cambodia	ERS014162	Cambodia	ERS025275	Cambodia	ERS032690	Cambodia	ERS071815
Cambodia	ERS014163	Cambodia	ERS025276	Cambodia	ERS032691	Cambodia	ERS071824
Cambodia	ERS014164	Cambodia	ERS028696	Cambodia	ERS032692	Cambodia	ERS088705

Cambodia	ERS014165	Cambodia	ERS028697	Cambodia	ERS032693	Cambodia	ERS088706
Cambodia	ERS014166	Cambodia	ERS028698	Cambodia	ERS032694	Cambodia	ERS140927
Cambodia	ERS140928	Cambodia	ERS174484	Cambodia	ERS010778	Ghana	ERS009734
Cambodia	ERS140935	Cambodia	ERS174485	Cambodia	ERS010782	Ghana	ERS010081
Cambodia	ERS140936	Cambodia	ERS174486	Cambodia	ERS010788	Ghana	ERS010083
Cambodia	ERS141406	Cambodia	ERS174488	Cambodia	ERS017561	Ghana	ERS010084
Cambodia	ERS141408	Cambodia	ERS174489	Cambodia	ERS024125	Ghana	ERS010085
Cambodia	ERS141414	Cambodia	ERS174492	Cambodia	ERS024126	Ghana	ERS010086
Cambodia	ERS141415	Cambodia	ERS174511	Cambodia	ERS024127	Ghana	ERS010087
Cambodia	ERS141417	Cambodia	ERS174512	Cambodia	ERS024128	Ghana	ERS010088
Cambodia	ERS141474	Cambodia	ERS174567	Cambodia	ERS024129	Ghana	ERS010089
Cambodia	ERS141480	Cambodia	ERS174569	Cambodia	ERS024130	Ghana	ERS010090
Cambodia	ERS141501	Cambodia	ERS174570	Cambodia	ERS024133	Ghana	ERS010124
Cambodia	ERS141502	Cambodia	ERS174571	Cambodia	ERS024135	Ghana	ERS010125
Cambodia	ERS142851	Cambodia	ERS174572	Cambodia	ERS024136	Ghana	ERS011021
Cambodia	ERS143415	Cambodia	ERS174573	Cambodia	ERS031999	Ghana	ERS011022
Cambodia	ERS143416	Cambodia	ERS174574	Cambodia	ERS032058	Ghana	ERS011023
Cambodia	ERS143417	Cambodia	ERS174575	Cambodia	ERS032071	Ghana	ERS011025
Cambodia	ERS143418	Cambodia	ERS174576	Cambodia	ERS032077	Ghana	ERS011026
Cambodia	ERS143420	Cambodia	ERS174577	Cambodia	ERS032082	Ghana	ERS011027
Cambodia	ERS143423	Cambodia	ERS174578	Cambodia	ERS032091	Ghana	ERS013064
Cambodia	ERS143425	Cambodia	ERS174580	Cambodia	ERS032093	Ghana	ERS013065
Cambodia	ERS143430	Cambodia	ERS175810	Cambodia	ERS032136	Ghana	ERS013066
Cambodia	ERS143432	Cambodia	ERS193641	Cambodia	ERS032637	Ghana	ERS013067
Cambodia	ERS143434	Cambodia	ERS199592	Cambodia	ERS010789	Ghana	ERS013068
Cambodia	ERS143436	Cambodia	ERS199597	Cambodia	ERS010790	Ghana	ERS013069
Cambodia	ERS143443	Cambodia	ERS199602	Cambodia	ERS024137	Ghana	ERS013071
Cambodia	ERS143444	Cambodia	ERS199607	Cambodia	ERS024139	Ghana	ERS013072
Cambodia	ERS143446	Cambodia	ERS199612	Cambodia	ERS024140	Ghana	ERS013073
Cambodia	ERS143447	Cambodia	ERS199617	Cambodia	ERS024142	Ghana	ERS013074
Cambodia	ERS143452	Cambodia	ERS199622	Cambodia	ERS024143	Ghana	ERS013075
Cambodia	ERS143453	Cambodia	ERS199627	Cambodia	ERS024144	Ghana	ERS013076
Cambodia	ERS143456	Cambodia	ERS199632	Cambodia	ERS024145	Ghana	ERS013077
Cambodia	ERS143457	Cambodia	ERS199637	Cambodia	ERS024146	Ghana	ERS013078
Cambodia	ERS143459	Cambodia	ERS224869	Cambodia	ERS024147	Ghana	ERS013079
Cambodia	ERS143461	Cambodia	ERS224889	Cambodia	ERS024148	Ghana	ERS013080
Cambodia	ERS143468	Cambodia	ERS224899	Cambodia	ERS024149	Ghana	ERS013081
Cambodia	ERS143470	Cambodia	ERS224904	Cambodia	ERS024150	Ghana	ERS013082
Cambodia	ERS143483	Cambodia	ERS224909	Cambodia	ERS024151	Ghana	ERS013091
Cambodia	ERS143486	Cambodia	ERS224914	Cambodia	ERS024152	Ghana	ERS013092
Cambodia	ERS143490	Cambodia	ERS336365	Cambodia	ERS032003	Ghana	ERS013093
Cambodia	ERS143491	Cambodia	ERS336376	Cambodia	ERS032017	Ghana	ERS013094
Cambodia	ERS143492	Cambodia	ERS024141	Cambodia	ERS032031	Ghana	ERS013095
Cambodia	ERS143500	Cambodia	ERS032014	Cambodia	ERS032039	Ghana	ERS013096
Cambodia	ERS143504	Cambodia	ERS009740	Cambodia	ERS032056	Ghana	ERS013097
Cambodia	ERS143512	Cambodia	ERS009744	Cambodia	ERS032106	Ghana	ERS013098
Cambodia	ERS143515	Cambodia	ERS009745	Cambodia	ERS032108	Ghana	ERS013099
Cambodia	ERS164616	Cambodia	ERS010054	Cambodia	ERS032638	Ghana	ERS013100
Cambodia	ERS164617	Cambodia	ERS010155	Cambodia	ERS157463	Ghana	ERS013101
Cambodia	ERS164619	Cambodia	ERS010156	Ghana	ERS009723	Ghana	ERS017384

Cambodia	ERS164622	Cambodia	ERS010670	Ghana	ERS009725	Ghana	ERS017385
Cambodia	ERS164623	Cambodia	ERS010671	Ghana	ERS009727	Ghana	ERS017386
Cambodia	ERS164624	Cambodia	ERS010776	Ghana	ERS009728	Ghana	ERS017387
Cambodia	ERS174483	Cambodia	ERS010777	Ghana	ERS009730	Ghana	ERS017388
Ghana	ERS017389	Ghana	ERS022963	Ghana	ERS032185	Malawi	ERS032653
Ghana	ERS017390	Ghana	ERS022964	Ghana	ERS032188	Malawi	ERS032654
Ghana	ERS017391	Ghana	ERS022965	Ghana	ERS032189	Malawi	ERS032657
Ghana	ERS017392	Ghana	ERS022966	Ghana	ERS032190	Malawi	ERS040098
Ghana	ERS017393	Ghana	ERS022967	Ghana	ERS032191	Malawi	ERS040099
Ghana	ERS017394	Ghana	ERS022968	Ghana	ERS032192	Malawi	ERS040100
Ghana	ERS017395	Ghana	ERS022969	Ghana	ERS032195	Malawi	ERS040101
Ghana	ERS017396	Ghana	ERS022970	Ghana	ERS032196	Malawi	ERS040103
Ghana	ERS017397	Ghana	ERS022971	Ghana	ERS032199	Malawi	ERS053866
Ghana	ERS017398	Ghana	ERS022972	Ghana	ERS032201	Malawi	ERS053871
Ghana	ERS017399	Ghana	ERS022973	Ghana	ERS032202	Malawi	ERS053875
Ghana	ERS017400	Ghana	ERS022974	Ghana	ERS032204	Malawi	ERS053876
Ghana	ERS017401	Ghana	ERS022975	Ghana	ERS032205	Malawi	ERS053877
Ghana	ERS017402	Ghana	ERS022976	Ghana	ERS032212	Malawi	ERS053938
Ghana	ERS022744	Ghana	ERS022977	Ghana	ERS032213	Malawi	ERS053940
Ghana	ERS022745	Ghana	ERS022978	Ghana	ERS032215	Malawi	ERS053942
Ghana	ERS022746	Ghana	ERS022979	Ghana	ERS032218	Malawi	ERS053944
Ghana	ERS022747	Ghana	ERS022981	Ghana	ERS032219	Malawi	ERS053945
Ghana	ERS022748	Ghana	ERS022982	Ghana	ERS032220	Malawi	ERS053947
Ghana	ERS022749	Ghana	ERS022984	Ghana	ERS032221	Malawi	ERS053948
Ghana	ERS022750	Ghana	ERS022986	Ghana	ERS032222	Malawi	ERS053949
Ghana	ERS022751	Ghana	ERS031998	Ghana	ERS032223	Malawi	ERS053950
Ghana	ERS022754	Ghana	ERS032001	Ghana	ERS032226	Malawi	ERS053952
Ghana	ERS022755	Ghana	ERS032002	Ghana	ERS032227	Malawi	ERS053953
Ghana	ERS022756	Ghana	ERS032007	Ghana	ERS032229	Malawi	ERS053954
Ghana	ERS022757	Ghana	ERS032011	Ghana	ERS032230	Malawi	ERS053955
Ghana	ERS022758	Ghana	ERS032012	Ghana	ERS032231	Malawi	ERS053956
Ghana	ERS022760	Ghana	ERS032028	Ghana	ERS032232	Malawi	ERS053957
Ghana	ERS022761	Ghana	ERS032030	Ghana	ERS032233	Malawi	ERS053958
Ghana	ERS022762	Ghana	ERS032032	Ghana	ERS032236	Malawi	ERS053960
Ghana	ERS022764	Ghana	ERS032033	Ghana	ERS032238	Malawi	ERS053878
Ghana	ERS022765	Ghana	ERS032034	Ghana	ERS032239	Malawi	ERS053880
Ghana	ERS022768	Ghana	ERS032035	Ghana	ERS032667	Malawi	ERS053890
Ghana	ERS022770	Ghana	ERS032038	Ghana	ERS032668	Malawi	ERS053891
Ghana	ERS022771	Ghana	ERS032044	Ghana	ERS032669	Malawi	ERS053895
Ghana	ERS022773	Ghana	ERS032047	Ghana	ERS032670	Malawi	ERS053897
Ghana	ERS022774	Ghana	ERS032053	Ghana	ERS032671	Malawi	ERS055901
Ghana	ERS022942	Ghana	ERS032054	Ghana	ERS032672	Malawi	ERS055903
Ghana	ERS022948	Ghana	ERS032067	Ghana	ERS032673	Malawi	ERS055904
Ghana	ERS022949	Ghana	ERS032170	Ghana	ERS032674	Malawi	ERS055905
Ghana	ERS022950	Ghana	ERS032171	Ghana	ERS032675	Malawi	ERS055906
Ghana	ERS022952	Ghana	ERS032172	Malawi	ERS032647	Malawi	ERS055907
Ghana	ERS022953	Ghana	ERS032173	Malawi	ERS032660	Malawi	ERS055908
Ghana	ERS022954	Ghana	ERS032174	Malawi	ERS032661	Malawi	ERS055909
Ghana	ERS022955	Ghana	ERS032176	Malawi	ERS032662	Malawi	ERS055911
Ghana	ERS022956	Ghana	ERS032177	Malawi	ERS032665	Malawi	ERS055913

Ghana	ERS022957	Ghana	ERS032178	Malawi	ERS032666	Malawi	ERS055914
Ghana	ERS022958	Ghana	ERS032180	Malawi	ERS032648	Malawi	ERS053902
Ghana	ERS022959	Ghana	ERS032181	Malawi	ERS032649	Malawi	ERS053903
Ghana	ERS022960	Ghana	ERS032182	Malawi	ERS032650	Malawi	ERS053904
Ghana	ERS022961	Ghana	ERS032183	Malawi	ERS032651	Malawi	ERS164642
Ghana	ERS022962	Ghana	ERS032184	Malawi	ERS032652	Malawi	ERS164677
Malawi	ERS164686	Malawi	ERS168607	Malawi	ERS188108	Malawi	ERS188141
Malawi	ERS168594	Malawi	ERS168608	Malawi	ERS188115	Malawi	ERS188148
Malawi	ERS168595	Malawi	ERS168609	Malawi	ERS188122	Thailand	ERS009703
Malawi	ERS168596	Malawi	ERS168610	Malawi	ERS188129	Thailand	ERS009705
Malawi	ERS168597	Malawi	ERS168611	Malawi	ERS188136	Thailand	ERS009706
Malawi	ERS168598	Malawi	ERS168612	Malawi	ERS188067	Thailand	ERS009707
Malawi	ERS168599	Malawi	ERS168614	Malawi	ERS188081	Thailand	ERS009709
Malawi	ERS168600	Malawi	ERS168615	Malawi	ERS188088	Thailand	ERS009710
Malawi	ERS168601	Malawi	ERS168616	Malawi	ERS188095	Thailand	ERS009713
Malawi	ERS168602	Malawi	ERS168617	Malawi	ERS188102	Thailand	ERS009714
Malawi	ERS168603	Malawi	ERS193667	Malawi	ERS188109	Thailand	ERS009715
Malawi	ERS168604	Malawi	ERS193672	Malawi	ERS188116	Thailand	ERS009716
Malawi	ERS168605	Malawi	ERS193623	Malawi	ERS188123	Thailand	ERS009717
Malawi	ERS168618	Malawi	ERS193628	Malawi	ERS188130	Thailand	ERS009718
Malawi	ERS168619	Malawi	ERS193638	Malawi	ERS188137	Thailand	ERS009722
Malawi	ERS168620	Malawi	ERS193643	Malawi	ERS188144	Thailand	ERS009956
Malawi	ERS168621	Malawi	ERS193648	Malawi	ERS188068	Thailand	ERS009957
Malawi	ERS168622	Malawi	ERS193653	Malawi	ERS188075	Thailand	ERS009958
Malawi	ERS168623	Malawi	ERS193658	Malawi	ERS188082	Thailand	ERS009959
Malawi	ERS168624	Malawi	ERS193663	Malawi	ERS188089	Thailand	ERS009960
Malawi	ERS168625	Malawi	ERS193668	Malawi	ERS188096	Thailand	ERS009961
Malawi	ERS168627	Malawi	ERS193673	Malawi	ERS188103	Thailand	ERS009962
Malawi	ERS168628	Malawi	ERS193678	Malawi	ERS188110	Thailand	ERS009963
Malawi	ERS168629	Malawi	ERS193624	Malawi	ERS188117	Thailand	ERS009964
Malawi	ERS168630	Malawi	ERS193629	Malawi	ERS188124	Thailand	ERS009968
Malawi	ERS168631	Malawi	ERS193634	Malawi	ERS188131	Thailand	ERS009969
Malawi	ERS168632	Malawi	ERS193639	Malawi	ERS188138	Thailand	ERS010141
Malawi	ERS168633	Malawi	ERS193644	Malawi	ERS188145	Thailand	ERS010190
Malawi	ERS168634	Malawi	ERS193654	Malawi	ERS188077	Thailand	ERS010308
Malawi	ERS168635	Malawi	ERS193659	Malawi	ERS188084	Thailand	ERS010314
Malawi	ERS168636	Malawi	ERS193664	Malawi	ERS188091	Thailand	ERS010349
Malawi	ERS168637	Malawi	ERS193669	Malawi	ERS188105	Thailand	ERS010353
Malawi	ERS168638	Malawi	ERS193674	Malawi	ERS188112	Thailand	ERS010478
Malawi	ERS168639	Malawi	ERS193679	Malawi	ERS188119	Thailand	ERS010514
Malawi	ERS168640	Malawi	ERS193625	Malawi	ERS188133	Thailand	ERS010522
Malawi	ERS168641	Malawi	ERS193630	Malawi	ERS188140	Thailand	ERS010524
Malawi	ERS168642	Malawi	ERS188069	Malawi	ERS188147	Thailand	ERS010525
Malawi	ERS168643	Malawi	ERS188076	Malawi	ERS188079	Thailand	ERS010526
Malawi	ERS168644	Malawi	ERS188090	Malawi	ERS188086	Thailand	ERS010528
Malawi	ERS168645	Malawi	ERS188097	Malawi	ERS188100	Thailand	ERS010530
Malawi	ERS168646	Malawi	ERS188111	Malawi	ERS188135	Thailand	ERS010531
Malawi	ERS168647	Malawi	ERS188118	Malawi	ERS188149	Thailand	ERS010532
Malawi	ERS168648	Malawi	ERS188125	Malawi	ERS188121	Thailand	ERS010600
Malawi	ERS168649	Malawi	ERS188132	Malawi	ERS188128	Thailand	ERS010601

Malawi	ERS168650	Malawi	ERS188139	Malawi	ERS188071	Thailand	ERS010602
Malawi	ERS168651	Malawi	ERS188146	Malawi	ERS188078	Thailand	ERS010605
Malawi	ERS168652	Malawi	ERS188066	Malawi	ERS188085	Thailand	ERS010606
Malawi	ERS168653	Malawi	ERS188073	Malawi	ERS188099	Thailand	ERS010622
Malawi	ERS175807	Malawi	ERS188080	Malawi	ERS188106	Thailand	ERS010626
Malawi	ERS175808	Malawi	ERS188087	Malawi	ERS188113	Thailand	ERS010634
Malawi	ERS175809	Malawi	ERS188094	Malawi	ERS188127	Thailand	ERS010648
Malawi	ERS168606	Malawi	ERS188101	Malawi	ERS188134	Thailand	ERS010649
Thailand	ERS010650	Thailand	ERS142879	Thailand	ERS174649	Vietnam	ERS143454
Thailand	ERS017464	Thailand	ERS142881	Thailand	ERS174651	Vietnam	ERS143462
Thailand	ERS017465	Thailand	ERS142883	Thailand	ERS174652	Vietnam	ERS143463
Thailand	ERS017466	Thailand	ERS142884	Thailand	ERS174653	Vietnam	ERS143464
Thailand	ERS017467	Thailand	ERS154522	Thailand	ERS174654	Vietnam	ERS143465
Thailand	ERS017468	Thailand	ERS164621	Thailand	ERS174655	Vietnam	ERS143467
Thailand	ERS017469	Thailand	ERS174521	Thailand	ERS174657	Vietnam	ERS143469
Thailand	ERS017470	Thailand	ERS174522	Thailand	ERS174658	Vietnam	ERS143471
Thailand	ERS017471	Thailand	ERS174523	Thailand	ERS174659	Vietnam	ERS143472
Thailand	ERS017472	Thailand	ERS174524	Thailand	ERS174661	Vietnam	ERS143473
Thailand	ERS023565	Thailand	ERS174525	Thailand	ERS174662	Vietnam	ERS143474
Thailand	ERS023566	Thailand	ERS174526	Thailand	ERS174663	Vietnam	ERS143475
Thailand	ERS023568	Thailand	ERS174527	Thailand	ERS174664	Vietnam	ERS143476
Thailand	ERS023569	Thailand	ERS174528	Thailand	ERS174665	Vietnam	ERS143477
Thailand	ERS023570	Thailand	ERS174529	Thailand	ERS174666	Vietnam	ERS143481
Thailand	ERS023572	Thailand	ERS174530	Thailand	ERS174667	Vietnam	ERS143484
Thailand	ERS023575	Thailand	ERS174531	Thailand	ERS174668	Vietnam	ERS143485
Thailand	ERS023576	Thailand	ERS174532	Thailand	ERS174669	Vietnam	ERS143493
Thailand	ERS142818	Thailand	ERS174533	Thailand	ERS347448	Vietnam	ERS143494
Thailand	ERS142819	Thailand	ERS174534	Thailand	ERS347472	Vietnam	ERS143495
Thailand	ERS142821	Thailand	ERS174535	Thailand	ERS347504	Vietnam	ERS143496
Thailand	ERS142822	Thailand	ERS174536	Thailand	ERS347512	Vietnam	ERS143497
Thailand	ERS142824	Thailand	ERS174537	Thailand	ERS010783	Vietnam	ERS143498
Thailand	ERS142825	Thailand	ERS174538	Thailand	ERS010784	Vietnam	ERS143499
Thailand	ERS142827	Thailand	ERS174539	Vietnam	ERS010034	Vietnam	ERS143501
Thailand	ERS142830	Thailand	ERS174540	Vietnam	ERS010035	Vietnam	ERS143502
Thailand	ERS142831	Thailand	ERS174541	Vietnam	ERS010036	Vietnam	ERS143505
Thailand	ERS142833	Thailand	ERS174631	Vietnam	ERS013083	Vietnam	ERS143506
Thailand	ERS142834	Thailand	ERS174632	Vietnam	ERS013084	Vietnam	ERS143508
Thailand	ERS142836	Thailand	ERS174633	Vietnam	ERS013085	Vietnam	ERS143509
Thailand	ERS142842	Thailand	ERS174634	Vietnam	ERS013086	Vietnam	ERS143511
Thailand	ERS142843	Thailand	ERS174635	Vietnam	ERS013087	Vietnam	ERS143514
Thailand	ERS142845	Thailand	ERS174636	Vietnam	ERS013088	Vietnam	ERS143516
Thailand	ERS142848	Thailand	ERS174637	Vietnam	ERS013089	Vietnam	ERS143518
Thailand	ERS142849	Thailand	ERS174638	Vietnam	ERS013102	Vietnam	ERS143519
Thailand	ERS142854	Thailand	ERS174639	Vietnam	ERS013103	Vietnam	ERS143520
Thailand	ERS142855	Thailand	ERS174640	Vietnam	ERS086846	Vietnam	ERS154466
Thailand	ERS142857	Thailand	ERS174641	Vietnam	ERS142875	Vietnam	ERS154483
Thailand	ERS142858	Thailand	ERS174642	Vietnam	ERS143419	Vietnam	ERS174506
Thailand	ERS142861	Thailand	ERS174643	Vietnam	ERS143421	Vietnam	ERS174542
Thailand	ERS142863	Thailand	ERS174644	Vietnam	ERS143424	Vietnam	ERS174543
Thailand	ERS142867	Thailand	ERS174645	Vietnam	ERS143428	Vietnam	ERS174544

Thailand	ERS142869	Thailand	ERS174646	Vietnam	ERS143429	Vietnam	ERS174545
Thailand	ERS142872	Thailand	ERS174647	Vietnam	ERS143433	Vietnam	ERS174546
Thailand	ERS142878	Thailand	ERS174648	Vietnam	ERS143437	Vietnam	ERS174547
Vietnam	ERS174548	Vietnam	ERS086810	Vietnam	ERS088710	Vietnam	ERS086997
Vietnam	ERS174550	Vietnam	ERS086811	Vietnam	ERS088712	Vietnam	ERS086998
Vietnam	ERS174551	Vietnam	ERS086812	Vietnam	ERS088713	Vietnam	ERS086999
Vietnam	ERS174552	Vietnam	ERS086814	Vietnam	ERS086797	Vietnam	ERS087000
Vietnam	ERS174553	Vietnam	ERS086815	Vietnam	ERS086798	Vietnam	ERS087028
Vietnam	ERS174554	Vietnam	ERS086816	Vietnam	ERS086799	Vietnam	ERS087029
Vietnam	ERS174555	Vietnam	ERS086817	Vietnam	ERS086800	Vietnam	ERS087030
Vietnam	ERS174556	Vietnam	ERS086819	Vietnam	ERS086801	Vietnam	ERS087032
Vietnam	ERS174557	Vietnam	ERS086820	Vietnam	ERS086802	Vietnam	ERS087033
Vietnam	ERS174558	Vietnam	ERS086821	Vietnam	ERS086803	Vietnam	ERS087034
Vietnam	ERS174560	Vietnam	ERS086822	Vietnam	ERS086804	Vietnam	ERS087035
Vietnam	ERS174670	Vietnam	ERS086823	Vietnam	ERS086806	Vietnam	ERS087036
Vietnam	ERS174671	Vietnam	ERS086824	Vietnam	ERS086807	Vietnam	ERS086886
Vietnam	ERS174672	Vietnam	ERS086825	Vietnam	ERS086808	Vietnam	ERS086887
Vietnam	ERS174673	Vietnam	ERS086827	Vietnam	ERS086809	Vietnam	ERS086888
Vietnam	ERS174674	Vietnam	ERS086828	Vietnam	ERS347506	Vietnam	ERS086909
Vietnam	ERS174675	Vietnam	ERS086833	Vietnam	ERS347521	Vietnam	ERS086913
Vietnam	ERS174676	Vietnam	ERS086834	Vietnam	ERS347529	Vietnam	ERS086934
Vietnam	ERS174677	Vietnam	ERS086836	Vietnam	ERS347537	Vietnam	ERS086950
Vietnam	ERS224919	Vietnam	ERS086837	Vietnam	ERS085458	Vietnam	ERS086981
Vietnam	ERS224924	Vietnam	ERS086839	Vietnam	ERS085459	Vietnam	ERS086983
Vietnam	ERS336368	Vietnam	ERS086840	Vietnam	ERS085460	Vietnam	ERS086984
Vietnam	ERS336375	Vietnam	ERS086847	Vietnam	ERS085461	Vietnam	ERS086985
Vietnam	ERS336380	Vietnam	ERS086848	Vietnam	ERS085462	Vietnam	ERS086986
Vietnam	ERS336381	Vietnam	ERS086864	Vietnam	ERS085463	Vietnam	ERS086987
Vietnam	ERS336386	Vietnam	ERS086865	Vietnam	ERS085464	Vietnam	ERS086991
Vietnam	ERS336392	Vietnam	ERS086872	Vietnam	ERS085465	Vietnam	ERS086994
Vietnam	ERS347474	Vietnam	ERS086873	Vietnam	ERS085466	Vietnam	ERS086995
Vietnam	ERS347475	Vietnam	ERS086874	Vietnam	ERS085467		
Vietnam	ERS347490	Vietnam	ERS086876	Vietnam	ERS085468		
Vietnam	ERS347499	Vietnam	ERS086884	Vietnam	ERS086796		

S5 Table. The 368 high-quality *Plasmodium vivax* isolates used in the study

Country	Identifier	Country	Identifier	Country	Identifier	Country	Identifier
Cambodia	ERR020103	Colombia	SRR1568159	Malaysia	ERR1138869	Mexico	SRR1568201
Cambodia	ERR023039	Colombia	SRR1568160	Malaysia	ERR1138870	Mexico	SRR1568218
Cambodia	ERR023040	Colombia	SRR1568169	Malaysia	ERR1138871	Mexico	SRR1568219
Cambodia	ERR023041	Colombia	SRR1568171	Malaysia	ERR1138872	Mexico	SRR1568223
Cambodia	ERR023042	Colombia	SRR1568207	Malaysia	ERR1138873	Mexico	SRR1568225
Cambodia	ERR027119	Colombia	SRR1568213	Malaysia	ERR1138875	Mexico	SRR1568231
Cambodia	ERR039234	Colombia	SRR1568221	Malaysia	ERR1138876	PNG	SRR1562605
Cambodia	ERR054080	Colombia	SRR1568227	Malaysia	ERR1138879	PNG	SRR1562669
Cambodia	ERR054082	Colombia	SRR1568230	Malaysia	ERR1138881	PNG	SRR1562672
Cambodia	ERR111729	Colombia	SRR1568235	Malaysia	ERR1138882	PNG	SRR1562960
Cambodia	ERR123849	Colombia	SRR1568236	Malaysia	ERR1138883	PNG	SRR1562963
Cambodia	ERR152408	Colombia	SRR1573226	Malaysia	ERR1138884	PNG	SRR1568105
Cambodia	ERR152410	Ethiopia	ERR925441	Malaysia	ERR1138885	PNG	SRR1568147
Cambodia	ERR152413	Ethiopia	ERR925440	Malaysia	ERR1475395	PNG	SRR1568177
Cambodia	ERR211549	Ethiopia	ERR925439	Malaysia	ERR1475396	PNG	SRR1568185
Cambodia	ERR211557	Ethiopia	ERR925438	Malaysia	ERR1475397	PNG	SRR1568189
Cambodia	ERR211561	Ethiopia	ERR925437	Malaysia	ERR1475398	PNG	SRR1568214
Cambodia	ERR216477	Ethiopia	ERR925436	Malaysia	ERR1475399	PNG	SRR1759411
Cambodia	ERR216554	Ethiopia	ERR925435	Malaysia	ERR1475418	PNG	SRR1759522
Cambodia	ERR337538	Ethiopia	ERR925434	Malaysia	ERR1475419	PNG	SRR1759523
Cambodia	ERR386533	Ethiopia	ERR925433	Malaysia	ERR1475420	PNG	SRR1759592
Cambodia	ERR386534	Ethiopia	ERR925431	Malaysia	ERR1475425	PNG	SRR1759594
Cambodia	ERR386535	Ethiopia	ERR925430	Malaysia	ERR1475427	PNG	ERR022864
Cambodia	ERR386536	Ethiopia	ERR925424	Malaysia	ERR1475429	PNG	ERR175552
Cambodia	ERR386537	Ethiopia	ERR925421	Malaysia	ERR1475430	PNG	ERR175555
Cambodia	ERR386538	Ethiopia	ERR925420	Malaysia	ERR1475434	PNG	ERR175557
Cambodia	ERR386539	Ethiopia	ERR925417	Malaysia	ERR1475439	PNG	ERR216469
Cambodia	ERR386541	Ethiopia	ERR925416	Malaysia	ERR1475441	PNG	ERR216474
Cambodia	ERR386542	Ethiopia	ERR925412	Malaysia	ERR1475451	PNG	ERR527450
Cambodia	ERR386543	Ethiopia	ERR925411	Malaysia	ERR1475456	PNG	ERR527453
Cambodia	ERR386546	Ethiopia	ERR925410	Malaysia	ERR1475457	PNG	ERR527467
Cambodia	ERR388742	Ethiopia	ERR925409	Malaysia	ERR054089	PNG	ERR527468
Colombia	SRR1562518	Ethiopia	ERR775192	Malaysia	ERR152414	Peru	SRR1562512
Colombia	SRR1562524	Ethiopia	ERR775191	Malaysia	ERR152415	Peru	SRR1562513
Colombia	SRR1562555	Ethiopia	ERR775190	Malaysia	ERR527337	Peru	SRR1562519
Colombia	SRR1562818	Ethiopia	ERR775189	Malaysia	ERR527363	Peru	SRR1562521
Colombia	SRR1562870	Malaysia	ERR1106842	Mexico	SRR1562522	Peru	SRR1562525
Colombia	SRR1562965	Malaysia	ERR1106843	Mexico	SRR1562526	Peru	SRR1562534
Colombia	SRR1562967	Malaysia	ERR1106846	Mexico	SRR1562839	Peru	SRR1562535
Colombia	SRR1562971	Malaysia	ERR1138855	Mexico	SRR1562840	Peru	SRR1562538
Colombia	SRR1562975	Malaysia	ERR1138856	Mexico	SRR1562968	Peru	SRR1562567
Colombia	SRR1564650	Malaysia	ERR1138857	Mexico	SRR1568077	Peru	SRR1562606
Colombia	SRR1564660	Malaysia	ERR1138858	Mexico	SRR1568110	Peru	SRR1562614
Colombia	SRR1564664	Malaysia	ERR1138861	Mexico	SRR1568126	Peru	SRR1562615
Colombia	SRR1564665	Malaysia	ERR1138862	Mexico	SRR1568127	Peru	SRR1562624

Colombia	SRR1564670	Malaysia	ERR1138864	Mexico	SRR1568150	Peru	SRR1562851
Colombia	SRR1568112	Malaysia	ERR1138865	Mexico	SRR1568153	Peru	SRR1562871
Colombia	SRR1568118	Malaysia	ERR1138866	Mexico	SRR1568158	Peru	SRR1562931
Colombia	SRR1568128	Malaysia	ERR1138867	Mexico	SRR1568181	Peru	SRR1562958
Colombia	SRR1568155	Malaysia	ERR1138868	Mexico	SRR1568190	Peru	SRR1562972
Peru	SRR1564630	Thailand	ERR527372	Thailand	ERR111714	Thailand	ERR337629
Peru	SRR1568107	Thailand	ERR527371	Thailand	ERR111715	Thailand	ERR527338
Peru	SRR1568113	Thailand	ERR527370	Thailand	ERR111716	Thailand	ERR527339
Peru	SRR1568117	Thailand	ERR527369	Thailand	ERR111717	Thailand	ERR527340
Peru	SRR1568122	Thailand	ERR527368	Thailand	ERR111718	Thailand	ERR527341
Peru	SRR1568123	Thailand	ERR527367	Thailand	ERR111719	Thailand	ERR527342
Peru	SRR1568149	Thailand	ERR527366	Thailand	ERR111720	Thailand	ERR527343
Peru	SRR1568157	Thailand	ERR527365	Thailand	ERR111721	Thailand	ERR527344
Peru	SRR1568162	Thailand	ERR527364	Thailand	ERR111722	Thailand	ERR527345
Peru	SRR1568163	Thailand	ERR426035	Thailand	ERR111723	Thailand	ERR527346
Peru	SRR1568165	Thailand	ERR426015	Thailand	ERR111724	Thailand	ERR527348
Peru	SRR1568166	Thailand	ERR404246	Thailand	ERR111725	Thailand	ERR527350
Peru	SRR1568168	Thailand	ERR164695	Thailand	ERR111726	Thailand	ERR527383
Peru	SRR1568172	Thailand	SRR1568229	Thailand	ERR111727	Thailand	ERR527382
Peru	SRR1568174	Thailand	SRR1568209	Thailand	ERR111728	Thailand	ERR527381
Peru	SRR1568175	Thailand	SRR1568208	Thailand	ERR1475350	Thailand	ERR527380
Peru	SRR1568178	Thailand	SRR1568205	Thailand	ERR1475351	Thailand	ERR527379
Peru	SRR1568179	Thailand	SRR1568186	Thailand	ERR1475352	Thailand	ERR527378
Peru	SRR1568182	Thailand	SRR1568180	Thailand	ERR1475353	Thailand	ERR527377
Peru	SRR1568183	Thailand	SRR1568161	Thailand	ERR1475354	Thailand	ERR527376
Peru	SRR1568184	Thailand	SRR1568154	Thailand	ERR1475355	Thailand	ERR527375
Peru	SRR1568187	Thailand	SRR1568152	Thailand	ERR337595	Thailand	ERR527374
Peru	SRR1568191	Thailand	SRR1568148	Thailand	ERR337596	Thailand	ERR527355
Peru	SRR1568195	Thailand	SRR1568109	Thailand	ERR337597	Thailand	ERR527354
Peru	SRR1568196	Thailand	SRR1568103	Thailand	ERR337599	Thailand	ERR527353
Peru	SRR1568198	Thailand	SRR1562974	Thailand	ERR337600	Thailand	ERR527352
Peru	SRR1568199	Thailand	SRR1562970	Thailand	ERR337601	Thailand	ERR527351
Peru	SRR1568202	Thailand	SRR1562962	Thailand	ERR337602	Thailand	ERR111709
Peru	SRR1568203	Thailand	SRR1562959	Thailand	ERR337603	Thailand	ERR111710
Peru	SRR1568206	Thailand	SRR1562845	Thailand	ERR337604	Thailand	ERR111711
Peru	SRR1568210	Thailand	SRR1562671	Thailand	ERR337605	Thailand	ERR111712
Peru	SRR1568211	Thailand	SRR1562616	Thailand	ERR337606	Thailand	ERR111713
Peru	SRR1568216	Thailand	SRR1562520	Thailand	ERR337607	Thailand	ERR337617
Peru	SRR1568232	Thailand	ERR773748	Thailand	ERR337608	Thailand	ERR337618
Peru	SRR1568234	Thailand	ERR773747	Thailand	ERR337609	Thailand	ERR337619
Peru	SRR1568787	Thailand	ERR773746	Thailand	ERR337610	Thailand	ERR337620
Peru	SRR1759047	Thailand	ERR773745	Thailand	ERR337611	Thailand	ERR337621
Peru	SRR1759122	Thailand	ERR713941	Thailand	ERR337612	Thailand	ERR337622
Peru	SRR1759307	Thailand	ERR527362	Thailand	ERR337613	Thailand	ERR337623
Peru	SRR1759336	Thailand	ERR527361	Thailand	ERR337614	Thailand	ERR337625
Thailand	ERR527385	Thailand	ERR527358	Thailand	ERR337615	Thailand	ERR337626
Thailand	ERR527384	Thailand	ERR527356	Thailand	ERR337616	Thailand	ERR337628

S6 Table
***Plasmodium falciparum* loci identified by *DeepSweep* (DS; with >3 SNPs)**

Chr.	Location	Gene ID (*PF3D7_*)	Gene Function	DS hits	iHS hits	Rsb hits
4	765952	0417400	conserved protein *(close to pfdhfr)*	8		37
5	852924	0520800	conserved protein	4		
5	921557	0522400	conserved protein *(close to pfmdr1)*	13		8
5	951346	0522900	zinc finger protein *(close to pfmdr1)*	8		
6	496916	0611800	conserved protein	6		
6	1109895	0627700	transportin	4		3
6	1115827	0627800	acetyl-CoA synthetase	20	11	39
6	1139634	0628100	HECT-domain (ubiquitin-transferase)	18	1	30
6	1163355	0628200	EIF2AK (PK4)	5		2
6	1292572	0630900	ATP-dependent RNA helicase HAS1	4		6
7	333558	0707200	conserved protein *(close to pfcrt)*	5		12
7	370246	0708000	cytoskeleton associated protein (close to *pfcrt)*	4		1
7	375694	0708200	conserved protein *(close to pfcrt)*	9		14
7	409992	0709100	Cg1 protein *(close to pfcrt)*	11		38
7	417927	0709300	Cg2 protein *(close to pfcrt)*	7		46
7	467220	0710200	conserved protein *(close to pfcrt)*	5	2	
8	488913	0809600	peptidase family C50 *(close to pfdhps)*	9	3	29
8	542388	0810600	ATP-dependent RNA helicase DBP1 (close to *pfdhps*)	5		8
8	563088	0811200	ER membrane protein complex subunit 1 (*close to pfdhps*)	7		11
8	585494	0811600	conserved protein *(close to pfdhps)*	4		
8	598114	0811900	RNA-binding protein *(close to pfdhps)*	6		4
12	750432	1219000	formin 2	4		1
12	943344	1223400	phospholipid-transporting ATPase	6		3
12	954302	1223500	conserved protein	8		11
14	2508460	1461800	conserved protein	5		
14	2536662	1462400	conserved protein	4		39

Chr, Chromosome; iHS and Rsb counts defined as the number of SNPs in a gene that have an |iHS| or |Rsb|score with a p-value < 0.0001; *pfdhfr* (Chr. 4: 749001*, pfmdr1* (Chr. 5: 960020), *pfcrt* (Chr. 7: 404770), *pfdhps* (Chr. 8: 549408)

S7 Table
***Plasmodium vivax* loci identified by *DeepSweep* (DS; with >3 SNPs)**

Chr.	Location	Gene ID (*PVP01_*)	Gene Function	DS Hits	iHS hits	Rsb hits
1	904054	0119600	Plasmodium exported protein	4		5
2	100527	0202000	hypothetical protein	11		4
2	156981	0203000	multidrug resistance-associated protein 1	7		33
2	745122	0217200	Plasmodium exported protein	5		
3	620559	0313900	exported serine/threonine protein kinase	4		
5	945918	0523400	Plasmodium exported protein (PHIST)	4		6
5	1041740	0525700	DNA helicase MCM9 *(close to pvdhfr)*	6		3
5	1047865	0525800	histone acetyltransferase *(close to pvdhfr)*	5	2	8
5	1064836	0526300	conserved protein *(close to pvdhfr)*	9	2	4
5	1070542	0526400	conserved protein *(close to pvdhfr)*	10	1	12
5	1093253	0526800	conserved protein *(close to pvdhfr)*	19	4	12
6	1011569	0624300	PIR protein	4		
7	64704	0701100	reticulocyte binding protein 1b	10		8
7	500160	0709800	cysteine repeat modular protein 1	11		0
7	1462407	0735200	Plasmodium exported protein	5		6
9	972542	0922400	peptidase M16	4		
9	1735229	0939900	RNA-binding protein	7	2	1
9	1752568	0940100	AP2 domain transcription factor	5		
9	2150596	0948800	tryptophan-rich protein	10		5
10	490336	1011000	zinc finger protein *(close to pvmdr1)*	11		1
10	1443984	1033900	tryptophan-rich protein	7		
10	1470845	1034400	Plasmodium exported protein	9		
11	61701	1101300	Plasmodium exported protein	14		2
12	3026696	1271500	lysophospholipase (PST-A)	8		
13	814038	1317300	conserved protein	7		15
14	43063	1401100	Plasmodium exported protein	5		
14	798622	1418100	AP2 domain transcription factor AP2-G3	7		1
14	1227470	1428700	conserved protein *(close to pvdhps)*	13	1	5
14	1232652	1428800	histone-arginine methyltransferase CARM1 *(close to pvdhps)*	4	1	
14	1245928	1429000	CCR4-associated factor 1 *(close to pvdhps)*	5		39
14	1300426	1430100	ABC1 family *(close to pvdhps)*	4		
14	1312634	1430400	JmjC domain-containing protein *(close to pvdhps)*	5	1	25
14	1320290	1430500	conserved protein *(close to pvdhps)*	4		
14	1336114	1430700	peptidase family C50	21		
14	1431856	1432900	GPI ethanolamine phosphate transferase 3	11	1	1

Chr, Chromosome; iHS and Rsb Counts defined as the number of SNPs in a gene that have an iHS or Rsb score with a p-value < 0.0001. *pvdhfr* (Chr. 5: 1078299), *pvmdr1* (Chr. 10: 480936, *pvdhps* (Chr. 14: 1271030)

S8 Table
***Plasmodium falciparum* loci with the most iHS and Rsb hits**

Chrom	Location	Gene ID (*PF3D7_*)	Function	iHS	Rsb	Deep Sweep
7	452987	0710000	conserved protein		49	2
7	417927	0709300	Cg2 protein		46	7
13	2116999	1352900	Plasmodium exported protein	1	41	
14	2536662	1462400	conserved protein		39	4
6	1115827	0627800	acetyl-CoA synthetase	11	39	20
7	409992	0709100	Cg1 protein		38	11
4	765952	0417400	conserved protein		37	8
5	1107081	0526600	conserved protein		37	
6	1139634	0628100	HECT-domain (ubiquitin-transferase)	1	30	18
8	488913	0809600	peptidase family C50	3	29	9
13	756296	1318300	conserved protein		25	
7	1379445	0731800	alpha/beta hydrolase		25	
14	3125133	1475900	KELT protein	4	24	
4	989562	0421700	conserved protein	20	23	
8	608343	0812100	proteasome activator complex subunit 4		20	1
11	1294496	1133400	apical membrane antigen 1	19	8	
10	1395940	1035200	S-antigen	18	8	
14	2792063	1468100	conserved protein		17	3
10	217522	1004600	conserved Plasmodium membrane protein		17	
5	1288394	0531500	unspecified product	1	17	
13	1011360	1324300	conserved Plasmodium membrane protein		17	
13	1421390	1335100	merozoite surface protein 7	2	16	
13	1466337	1335900	thrombospondin-related anonymous protein	16	12	
7	340301	0707300	rhoptry-associated membrane antigen		16	1
5	1036670	0525000	zinc finger protein		16	
12	785829	1219600	aminophospholipid-transporting P-ATPase	15		
9	285998	0905700	autophagy-related protein 3		15	
7	929713	0721500	conserved Plasmodium membrane protein		15	
8	427868	0808500	Plasmodium RNA of unknown function RUF6	2	15	
8	549345	0810800	HPPK-DHPS		15	4
8	1313279	0830800	SURFIN 8.2	15	4	
7	375693	0708200	conserved protein		14	9
5	482369	0511400	conserved protein	2	14	
7	951490	0722300	ubiquitin carboxyl-terminal hydrolase		13	1
8	919102	0820300	conserved protein		13	
14	2527372	1462300	GTP-binding protein		13	2
7	333557	0707200	conserved protein		12	5
3	221968	0304600	circumsporozoite (CS) protein	1	12	
2	618524	0215000	acyl-CoA synthetase		12	
6	1265574	0630300	DNA polymerase epsilon catalytic subunit A	1	11	1
13	108319	1301900	Plasmodium exported protein	1	11	
3	653725	0316200	conserved protein		11	
13	479886	1311100	meiosis-specific nuclear structural protein 1		11	
4	695218	0415800	RING zinc finger protein		11	

6	852589	0620400	merozoite surface protein 10		11	
7	382171	0708400	heat shock protein 90		11	
6	590882	0614100	conserved protein		11	
8	563087	0811200	ER membrane protein complex subunit 1		11	7
12	2119088	1252100	rhoptry neck protein 3		11	
12	954302	1223500	conserved protein		11	8
5	64553	0501200	parasite-infected erythrocyte surface protein		11	
9	281899	0905600	WD repeat-containing protein 66		11	
10	1400721	1035300	glutamate-rich protein GLURP	10	1	
5	1042329	0525100	acyl-CoA synthetase		10	
8	846031	0818600	BEM46-like protein	2	10	
5	1184595	0528900	conserved protein		10	
6	665485	0615900	protein phosphatase		10	
7	432013	0709600	ribonucleases P/MRP protein subunit POP1		9	
12	175563	1203300	conserved protein		9	
6	571507	0613800	AP2 domain transcription factor		9	
7	883783	0720400	apoptosis-inducing factor		9	
14	411037	1410300	WD repeat-containing protein		9	
10	1573998	1039000	serine/threonine protein kinase, FIKK family		9	
11	138792	1103000	conserved protein		9	
13	2341158	1359000	conserved protein		9	
10	61898	1001000	Plasmodium exported protein (hyp12)	2	9	
13	2386189	1359700	conserved protein		9	
12	712688	1218200	conserved protein		9	
11	1376532	1135100	protein phosphatase PPM8		9	
6	241806	0605800	DNA repair protein RAD50		9	
13	100779	1301700	CX3CL1-binding protein 2	9	2	
7	404151	0709000	chloroquine resistance transporter		8	4
10	1420620	1035800	probable protein		8	
14	1990103	1448500	conserved protein		8	
5	921557	0522400	conserved protein		8	13
14	3121488	1475800	conserved protein	8	5	
7	1089266	0726000	28S ribosomal RNA		8	
12	660272	1216600	CelTOS	6	8	
13	780617	1318900	conserved protein		8	
10	497315	1012900	autophagy-related protein 18		8	
6	1272955	0630400	conserved protein		8	
8	542387	0810600	ATP-dependent RNA helicase DBP1		8	5
7	505906	0711500	regulator of chromosome condensation	7	8	3
7	729631	0716700	conserved protein		8	
8	555489	0811000	cullin-1		8	2
14	46645	1401200	Plasmodium exported protein		8	
9	1437390	0936300	ring-exported protein 3		8	
2	501713	0212400	conserved Plasmodium membrane protein		7	
7	932493	0721600	40S ribosomal protein S5		7	
9	1416021	0935800	cytoadherence linked asexual protein 9		7	
2	855072	0221200	Plasmodium exported protein (hyp15)		7	
14	1226553	1431200	OST-HTH associated domain protein		7	
2	109622	0202100	liver stage associated protein 2		7	

8	331678	0806100	conserved protein	7	
14	2183066	1453200	conserved protein	7	1
7	957542	0722500	pre-mRNA-splicing factor CWC15	7	
14	304487	1408200	AP2 domain transcription factor AP2-G2	7	
9	110619	0902500	serine/threonine protein kinase, FIKK family	6	
9	747006	0918100	cytochrome b5-like heme/steroid binding protein	6	

iHS and Rsb Counts defined as the number of SNPs in a gene that have an iHS or Rsb score with a p-value < 0.0001

S9 Table
***Plasmodium vivax* loci with the most iHS and Rsb hits**

Chr.	Location	Gene ID (*PVP01_*)	Function	iHS hits	Rsb hits	Deep Sweep
2	148495	0202900	18S ribosomal RNA	2	62	
10	189242	1003700	phosphoenolpyruvate/phosphate translocator	2	45	
14	1245928	1429000	CCR4-associated factor 1		39	5
2	156981	0203000	multidrug resistance-associated protein 1		33	7
14	1284723	1429800	protein phosphatase PPM7		29	
10	146870	1002700	conserved protein	2	28	3
10	153038	1002800	SURF1 domain-containing protein		26	
14	1312633	1430400	JmjC domain-containing protein	1	25	5
10	184519	1003600	conserved protein		24	
1	883223	0119200	Plasmodium exported protein (PHISTc)		21	
10	318571	1007200	conserved protein	5	18	
10	205509	1004100	conserved protein		18	
3	123931	0302600	conserved protein	13	16	
10	1376089	1032000	50S ribosomal protein L28, apicoplast	6	16	
13	814038	1317300	conserved protein		15	7
14	2257490	1451700	asparagine and aspartate rich protein 1		13	
14	1263931	1429300	cullin-1		13	2
5	210653	0504700	18S ribosomal RNA		12	
11	1483167	1134800	major facilitator superfamily-related transporter		12	
14	823643	1418900	conserved protein		12	
10	1324328	1030700	hypothetical protein		12	2
12	788337	1219200	hypothetical protein	8	12	2
7	551240	0711200	tRNA (adenine(58)-N(1))-methyltransferase non- catalytic subunit TRM6	1	12	
14	1255147	1429100	ER membrane protein complex subunit 1		12	3
5	1070542	0526400	conserved protein	1	12	10
2	105832	0202100	Plasmodium exported protein	2	12	
14	1267716	1429400	conserved protein		12	1
2	175717	0203400	eukaryotic translation initiation factor 4E	2	12	2
5	1093253	0526800	conserved protein	4	12	19
8	108099	0802000	5.8S ribosomal RNA		11	
9	328701	0905600	RNA polymerase subunit	1	11	
10	168725	1003200	conserved protein		11	
10	1387953	1032500	conserved protein	1	11	
13	2014020	1346200	ribosomal protein S27a	2	11	
4	212211	0404700	Plasmodium exported protein		10	
13	2017523	1346400	zinc finger protein	9	10	
4	584464	0414300	conserved protein		10	
5	1258182	0529800	AP2 domain transcription factor		10	
14	2696171	1462600	conserved protein	1	10	
7	1217570	0728900	merozoite surface protein 1	10	5	1
14	2207640	1450700	CG2-related protein		10	
14	1278613	1429700	ATP-dependent RNA helicase DBP1		10	

10	159076	1002900	formin 1		10	
14	2035601	1447000	WD repeat-containing protein	1	10	
12	2248858	1255000	rhoptry neck protein 2		9	2
6	456433	0610300	glutamine--fructose-6-phosphate aminotransferase [isomerizing]		9	
11	1742196	1141100	conserved protein		9	
5	1073823	0526500	mRNA-binding protein PUF2		9	2
11	1675817	1139500	cardiolipin synthetase		9	
10	521068	1011500	conserved protein		9	3
12	2987482	1270700	conserved protein	1	9	
4	680716	0416700	conserved protein		9	
4	747321	0418000	serine-repeat antigen (SERA)		9	
5	190906	0504400	sporozoite invasion-associated protein 2		9	
11	698237	1116200	NLI interacting factor-like phosphatase		9	
5	855549	0520900	conserved protein	1	9	
14	2710458	1462900	ankyrin-repeat protein		8	
11	1423420	1133300	conserved oligomeric Golgi complex subunit 4		8	
5	1077709	0526600	bifunctional dihydrofolate reductase-thymidylate synthase		8	5
6	579569	0613600	partial CSTF domain-containing protein		8	
8	1611934	0838000	Plasmodium exported protein		8	
7	64704	0701100	reticulocyte binding protein 1b		8	10
7	692112	0715200	conserved protein		8	
13	567027	1312400	conserved protein		8	
5	1368574	0532400	cysteine-rich protective antigen	8	2	
12	324002	1208000	6-cysteine protein	1	8	
12	153028	1203700	conserved protein		8	
5	1047865	0525800	histone acetyltransferase	2	8	5
2	391828	0209400	conserved protein		8	
3	496884	0311200	conserved protein		7	
1	772847	0117000	filament assembling protein		7	
14	1260196	1429200	mitochondrial carrier protein		7	1
14	1270993	1429500	hydroxymethyldihydropterin pyrophosphokinase-dihydropteroate synthase		7	4
14	2701487	1462700	kinesin		7	
9	274077	0904400	actin-like protein	2	7	
8	1504916	0835500	conserved protein		7	
11	1247714	1128700	tRNA Alanine		7	
6	668362	0616000	ookinete surface protein P28	1	7	
10	449901	1010100	divalent metal transporter		7	1
10	1327562	1030800	hypothetical protein		7	1
10	1306926	1030300	myosin B		7	
10	374884	1008500	conserved protein		7	
14	815587	1418600	conserved protein		7	
10	1407703	1033100	conserved protein	7	0	
14	817482	1418700	40S ribosomal protein S19		7	
10	1303077	1030200	60S ribosomal protein L31		7	
6	283136	0606900	conserved protein		6	
11	141431	1103200	DNA repair endonuclease XPF		6	

7	1140161	0726700	conserved protein		6	
11	678373	1115800	conserved protein		6	
12	768965	1218700	thrombospondin-related anonymous protein		6	
7	76205	0701200	reticulocyte binding protein 1a	5	6	
11	1629987	1138400	transketolase		6	
13	1291399	1330800	liver specific protein 1		6	1
7	1462407	0735200	Plasmodium exported protein		6	5
6	427598	0609500	ATP synthase-associated protein		6	
13	2015443	1346300	small nucleolar RNA snoR22		6	
5	722009	0517200	zinc finger protein		6	
13	1139543	1326200	actin-related protein	2	6	
5	945918	0523400	Plasmodium exported protein (PHIST)		6	4

iHS and Rsb Counts defined as the number of SNPs in a gene that have an iHS or Rsb score with a p-value < 0.0001

S1 Figure
The creation of haplo-images

Panel 1)

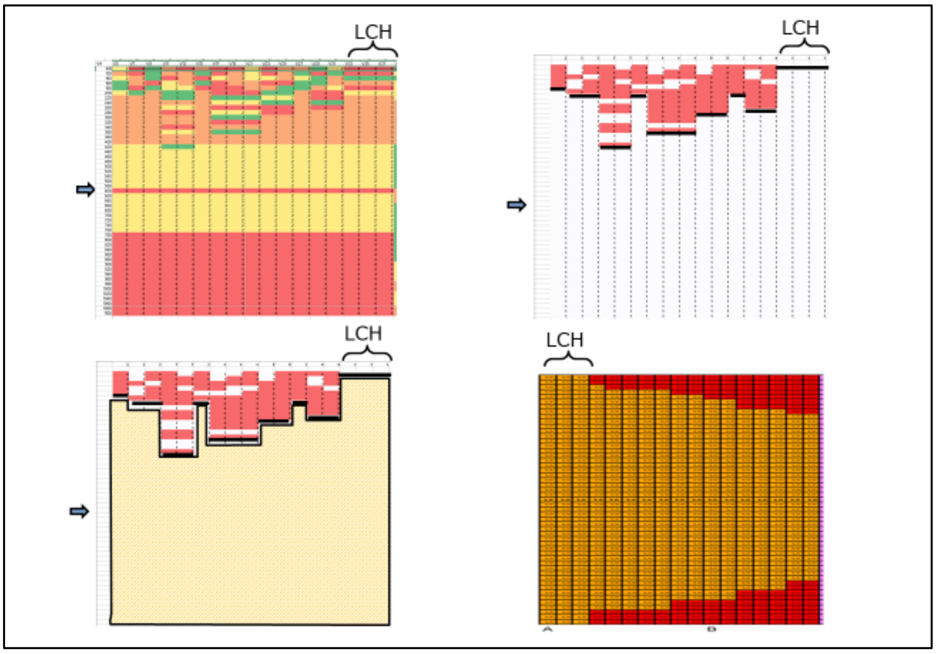

Panel 2)

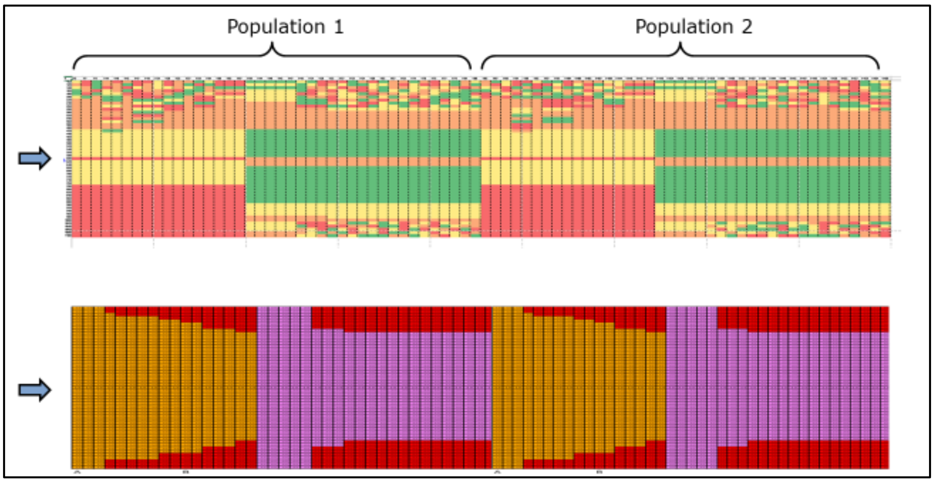

Panel 1 Top-left: The image shows a small hypothetical genomic dataset of 51 SNPs (rows) and 17 samples (columns) with nucleotides re-coded as 1,2,3 and 4. All samples come from one population and have the same allele in the mid-position (row 25, highlighted with arrow). The creation of a haplo-image for this 25th SNP would start with determining the longest common haplotype (LCH). In this example, the last three columns share the LCH. **Panel 1 Top-right:** The differences between these three samples that make up the LCH and the other samples are shown in red. Panel 1 Bottom-left: The overlaps between the LCH samples and the other samples is shown in yellow. **Panel 1 Bottom-right:** A re-ordering based on shared overlap gives a haplo-image (for one population and one allele). **Panel 2 Top**: A hypothetical dataset with two alleles and two populations. **Panel 2 Bottom:** The resulting haplo-image. It should be noted that the actual process involves haplo-images each comprised of 1,401 SNPs. These SNPs were however not adjacent to one another but were chosen to be a specific distance apart. This distance was 100 basepoints for *P. falciparum* and 50 basepoints for *P. vivax*. The resulting genomic haplotype matrices have a size equivalent to the range of SNPs (e.g. 1,401 SNPs) and the number of samples (e.g. 1,100 samples). However, to improve the computational speed of the *DeepSweep* algorithm, these genomic matrices were further shrunk to a size of a height of 40 pixels by a width of 200 pixels, using "nearest" interpolation in the Scipy image package (59).

S2 Figure

Workflow

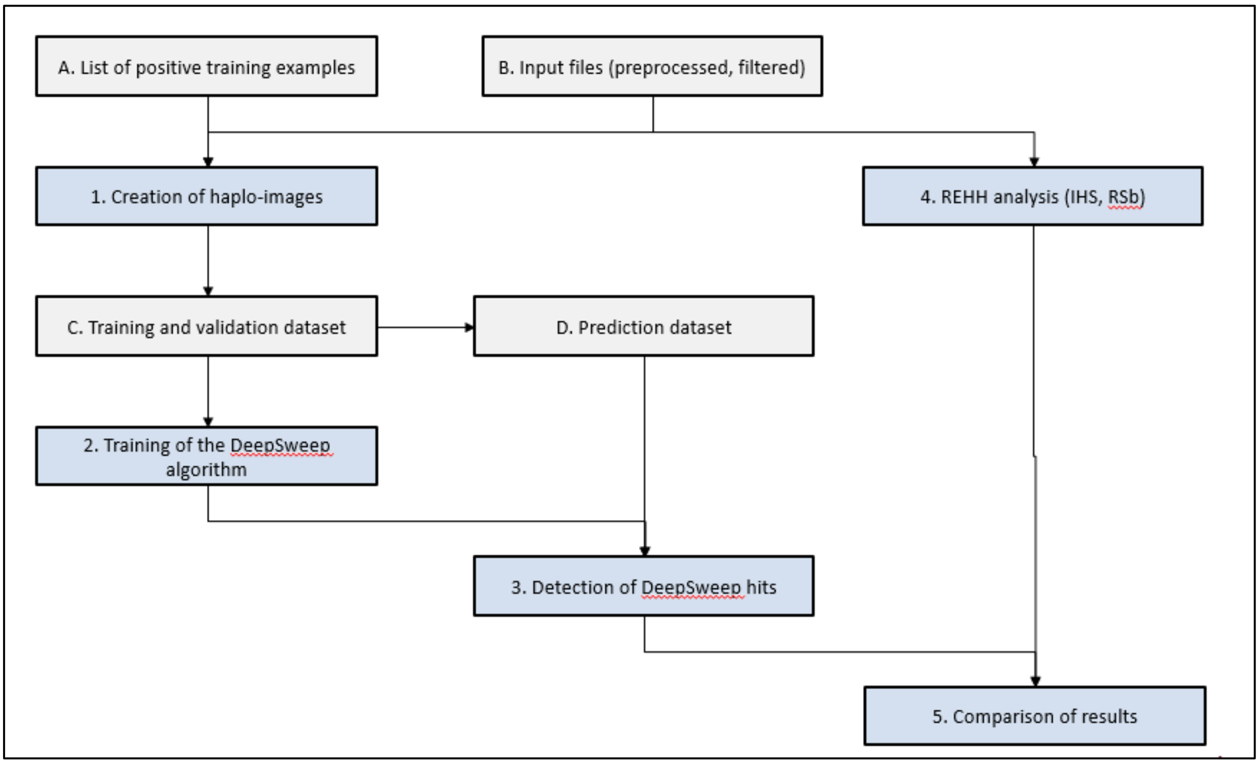

Legend: Grey boxes are datasets, blue boxes are activity steps. Step 1 (creation of haplo-images) is further expanded upon in **S1 Figure**.

S3 Figure

Exemplar images of simulated isolates undergoing different types of sweeps or neutral evolution

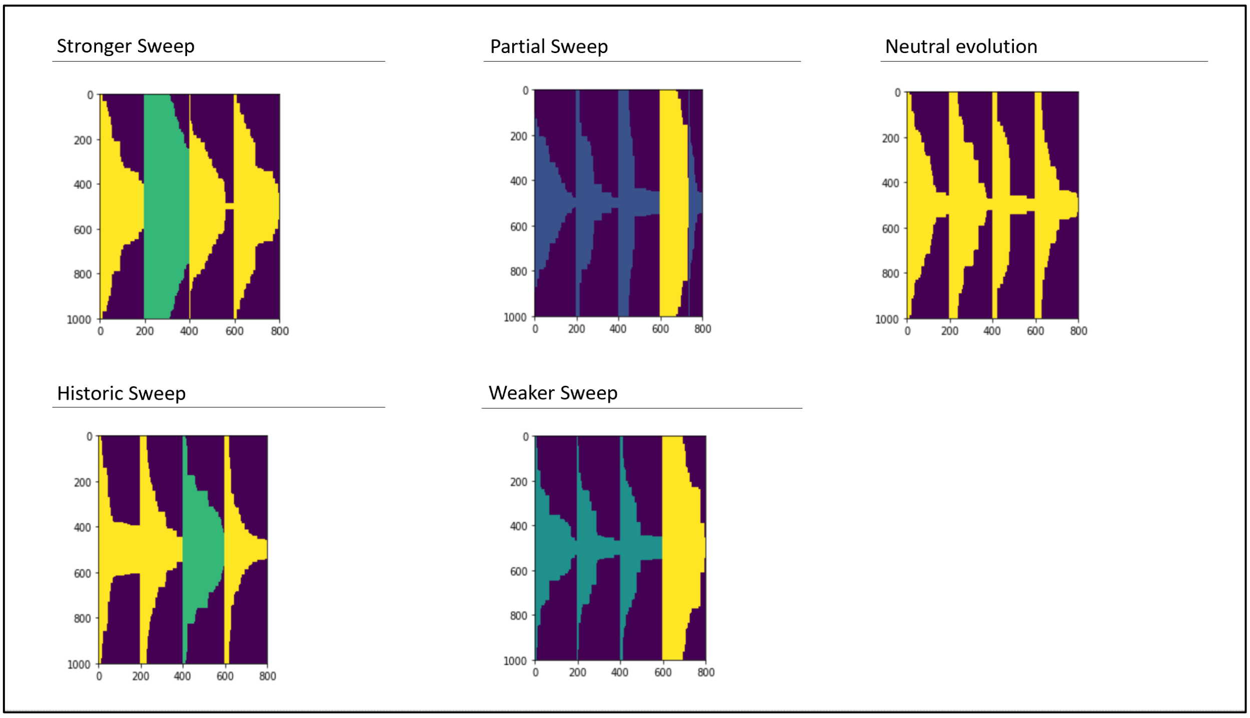

Each individual diagram is a haplo-image of a specific SNP for population of parasites, with the genomic information simulated following the settings as described in **S1 Table**. The haplo-images are created following the explanation in **S1 Figure**, with genomes of the individual parasites ordered on the horizontal axis and the overlap in haplotype for the SNP in focus shown on the vertical axis. The colour coding links to overlap in specific nucleotides (yellow, green, dark blue, light blue) with the purple background indicating no overlap. The sweeps in these illustrative examples are different nucleotides/alleles than the ancestral nucleotide/allele. Weaker sweep refers to simulation with relatively low selection coefficient; historic refers to a simulated sweep that occurred further in the past; partial refers to a partial sweep that is not fully fixed; stronger refers to a sweep with a relatively high selection coefficient.

S4 Figure

Model performance on simulated datasets

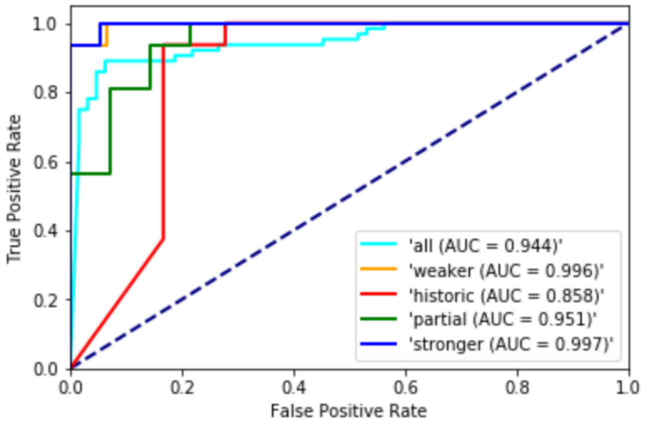

“weaker” refers to the simulation with a low selection coefficient; “historic” refers to a simulated sweep that occurred further in the past; “partial” refers to a partial sweep that is not fully fixed; “stronger” refers to a sweep with a high selection coefficient; “all” refers to all sweeps combined; AUC Area under the ROC Curve.

S5 Figure

Distribution of the minor allele frequencies across the SNPs

a) ***P. falciparum*** **(N=750k SNPs)**

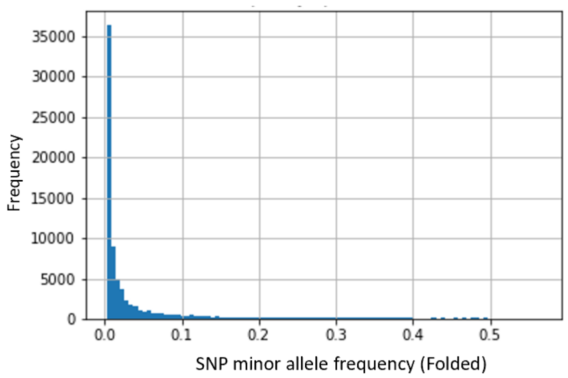

b) ***P. vivax*** **(N=588k SNPs)**

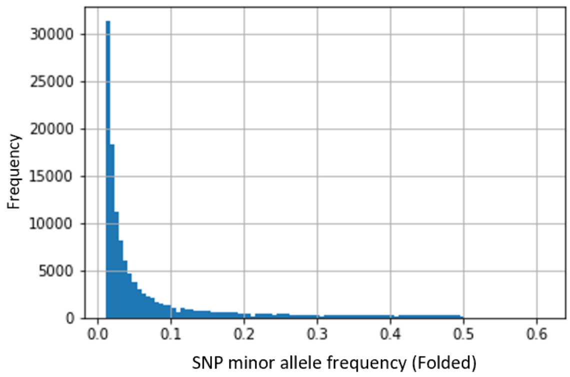

S6 Figure

Model performance for *Plasmodium falciparum* and *P. vivax* on training and validation datasets

Plasmodium falciparum	*Plasmodium vivax*
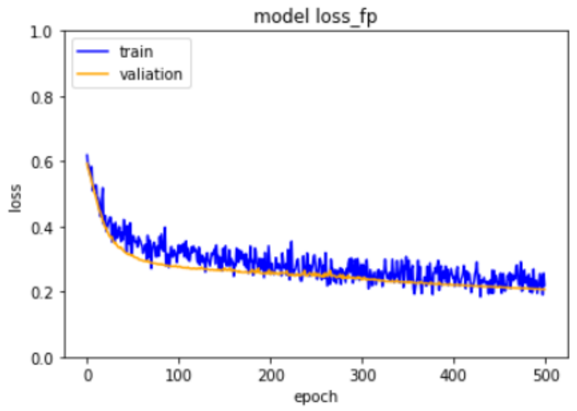	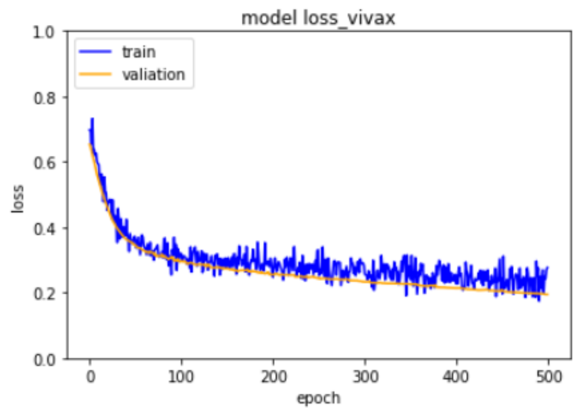
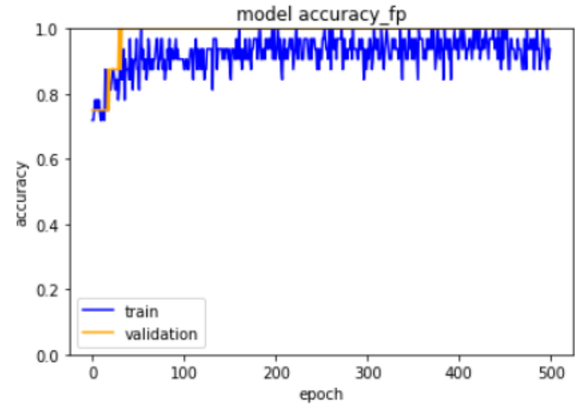	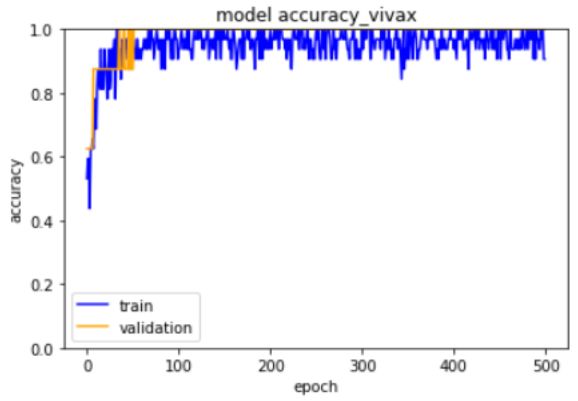

The left panel shows the performance of the model in the *P. falciparum* parasite data, and the right panel shows the performance of the model in the *P. vivax* parasite data. The top panel shows model loss (measured as binomial loss) and the bottom panel shows model accuracy (measured as correct classification). The blue lines show the statistics for the training datasets and the orange lines show the statistics for the validation datasets. A negative slope in the top panel indicates a decrease in loss as the model trains over more epochs. An increasing slope in the bottom panel indicates an increase in accuracy and a reduction in misclassification as the model trains over more epochs.

S7 Figure

Relationship between -log10 p-value of Rsb hits and number of *DeepSweep* hits

a) ***P. falciparum***

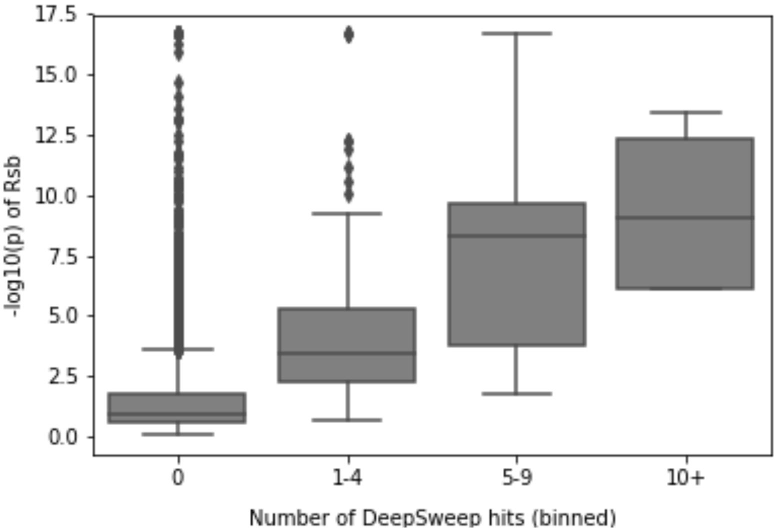

b) ***P. vivax***

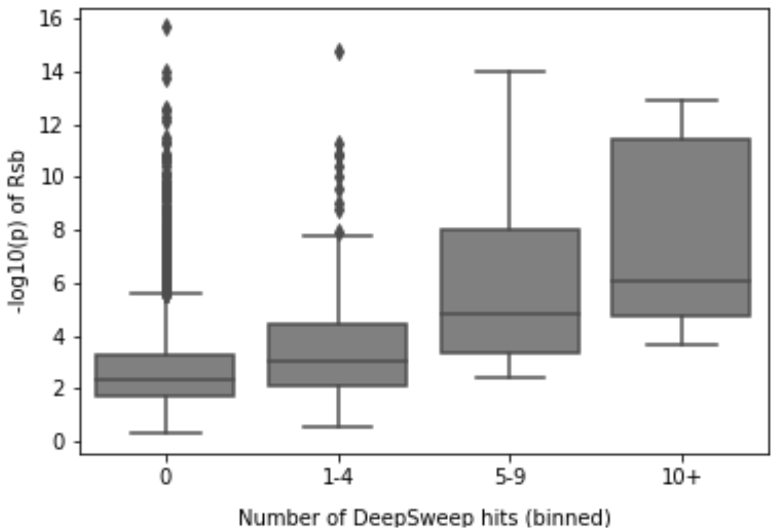

Supplement: Supplementary file 1 — Additional file 1: Table S1. Simulation parameters for the data generation using SFS_Code software. Table S2. Performance of Convolutional Neural Network (CNN) model structures on simulated datasets. Table S3. Sample origin by geographic location. Table S4. The 1,125 high-quality P. falciparum isolates used in this study. Table S5. The 368 high-quality P. vivax isolates used in the study. Table S6. Plasmodium falciparum loci identified by DeepSweep (DS; with >3 SNPs). Table S7. Plasmodium vivax loci identified by DeepSweep (DS; with >3 SNPs). Table S8. Plasmodium falciparum loci with the most iHS and Rsb hits. Table S9. Plasmodium vivax loci with the most iHS and Rsb hits. Figure S1. The creation of haplo-images. Figure S2. Workflow. Figure S3. Exemplar images of simulated isolates undergoing different types of sweeps or neutral evolution. Figure S4. Model performance on simulated datasets. Figure S5. Distribution of the minor allele frequencies across the SNPs. Figure S6. Model performance for Plasmodium falciparum and P. vivax on training and validation datasets. Figure S7. Relationship between -log10 p-value of Rsb hits and number of DeepSweep hits. [file 12936_2021_3788_MOESM1_ESM.pdf]
